# Potential scalp acupuncture and brain stimulation targets for common neurological disorders: evidence from neuroimaging studies

**DOI:** 10.1186/s13020-025-01106-0

**Published:** 2025-05-07

**Authors:** Yuefeng Wu, Qiao Kong, Yuanyuan Li, Yuan Feng, Binlong Zhang, Yu Liu, Siyi Yu, Jiao Liu, Jin Cao, Fangyuan Cui, Jian Kong

**Affiliations:** 1 Department of Psychiatry, Massachusetts General Hospital, Harvard Medical School, Boston, MA 02129 USA; 2 Guang’anmen Hospital, China Academy of Chinese Medical Science, Beijing, 100053 China; 3 Acupuncture-Moxibustion and Tuina School, Chengdu University of Traditional Chinese Medicine, Chengdu, 611137 China; 4 College of Traditional Chinese Medicine, Capital Medical University, 100000 Beijing, China; 5 School of Life Sciences, Beijing University of Chinese Medicine, Beijing, 100029 China; 6 Dongzhimen Hospital, Beijing University of Chinese Medicine, Beijing, 100700 China

**Keywords:** Scalp acupuncture, Neuromodulation, Neuroimaging, Neurological disorders, Brain stimulation

## Abstract

**Background:**

Scalp acupuncture is a promising potential therapy for neurological disorders. However, the development of its stimulation targets—both in identifying novel targets and refining the precision of their localization—has advanced slowly, largely due to the inadequate integration of brain science findings. This study leverages advances in brain neuroimaging to identify evidence-based cortical targets, enhancing the potential of scalp acupuncture and other brain stimulation techniques.

**Methods:**

Using the Neurosynth Compose platform, systematic meta-analyses of neuroimaging studies were conducted to identify potential surface cortical targets for ten neurological conditions: Subjective Cognitive Decline (SCD), Mild Cognitive Impairment (MCI), Alzheimer’s Disease (AD), Parkinson’s Disease (PD), Multiple System Atrophy (MSA), Post-Stroke Aphasia (PSA), Primary Progressive Aphasia (PPA), Dyslexia, Chronic Pain, and Disorders of Consciousness (DoC). These targets were projected onto the scalp, further localized using scalp acupuncture lines, traditional acupoints and EEG 10–20 system.

**Results:**

We have identified specific cortical targets for scalp acupuncture associated with ten neurological disorders. Our findings are broadly consistent with current scalp acupuncture protocols while introducing additional new stimulation targets, such as the inferior temporal gyrus for memory processing and the angular gyrus for visuospatial attention. Additionally, the identified targets align with evidence from non-invasive brain stimulation, supporting therapeutic strategies for conditions such as movement disorders and cognitive impairments by targeting areas like the dorsolateral prefrontal cortex and primary motor cortex.

**Conclusion:**

Our findings provide a foundation for developing a brain imaging-based scalp acupuncture protocol for neurological disorders. The identified targets may also be used as brain stimulation targets for these disorders.

**Supplementary Information:**

The online version contains supplementary material available at 10.1186/s13020-025-01106-0.

## Background

Scalp acupuncture is a therapeutic approach that involves the insertion of needles into specific scalp regions to address a variety of medical conditions [1]. Through targeted stimulation of corresponding cortical regions, scalp acupuncture can influence neural pathways and brain function, providing relief for these conditions [2]. Both the World Health Organization (WHO) and Chinese authorities have established guidelines for scalp acupuncture, laid the groundwork for its clinical application.

These standards, however, primarily focus on anatomical positioning and the indications for stimulation lines without incorporating advanced findings from neuroimaging studies [3]. Most importantly, current standards do not specify disease-specific scalp acupuncture targets or protocols that are crucial for the clinical application of the promising intervention.

Neuroimaging technologies are integral to contemporary brain science research, providing insights into structural and functional brain alterations associated with neurological disorders. These technologies not only aid in diagnosis and prognosis but also enable the assessment of therapeutic interventions. Non-invasive brain stimulation (NIBS) techniques—such as repetitive transcranial magnetic stimulation (rTMS) and transcranial direct current stimulation (tDCS)—similarly provide therapeutic effects by modulating cortical excitability in targeted brain regions. Experimental evidence suggests that such stimulation can improve cognitive and motor functions, reduce depressive symptoms, and enhance quality of life across a range of neurological disorders [4–6]. These improvements highlight a potential role for similar targeting methods in scalp acupuncture, as both approaches rely on accurate locations / targets of stimulation.

Given these parallels, the selection of stimulation targets in scalp acupuncture could be optimized by integrating findings from neuroimaging. Neuroimaging-based selection strategies could refine scalp acupuncture techniques by pinpointing specific brain regions / locations associated with particular symptoms or disorders, thereby enhancing clinical efficacy. Building on these concepts, our previous research [7–9] utilized the Neurosynth platform to automatically identify brain region clusters associated with various neurological disorders, introducing a novel approach to linking brain imaging data with therapeutic targets in acupuncture.

However, the methodological limitations of Neurosynth, notably its inability to manually exclude manuscripts or tables, have imposed constraints on our previous findings.

Following the recent enhancement of the Neurosynth platform to Neurosynth Compose, which includes an expanded repository of publications and advanced neuroimaging meta-analysis tools, and a more robust foundation has been established for identifying disease-specific targets. This enhancement allows for greater flexibility and precision in selecting and analyzing a broader range of neurological diseases. Building on these improved capabilities, this study revisits and refines our previous findings under the title “Targets for Common Neurological Disorders”, leveraging the platform’s advanced functionalities for a more comprehensive analysis.

In this study, we leverage the Neurosynth Compose neuroimaging literature analysis platform to identify potential scalp acupuncture targets across prevalent neurological disorders. Specifically, we performed the meta-analysis on ten disorders, i.e., subjective cognitive decline (SCD), mild cognitive impairment (MCI), Alzheimer’s disease (AD), Parkinson’s disease (PD), multiple system atrophy (MSA), primary progressive aphasia (PPA), post-stroke aphasia (PSA), dyslexia, chronic pain, and disorders of consciousness (DoC). For each disorder, we identify 3–9 potential stimulation targets, offering new insights and practical guidance to support evidence-based, condition-specific scalp acupuncture treatment, and advancing its application in clinical practice. In addition, the identified targets can be applied to other brain stimulation techniques, such as tDCS and rTMS.

## Methods

This study aims to explore potential scalp acupuncture targets for common primary neurological diseases, categorized as neurodegenerative diseases (e.g., SCD, MCI, AD), movement disorders (e.g., PD, MSA), language and language-related diseases (e.g., PSA, PPA, Dyslexia,), and other common neurological symptoms (e.g., Chronic Pain, DoC). A novel data search and statistical analysis platform, Neurosynth Compose, was used to identify brain surface targets through a systematic meta-analysis of extensive literature. Based on the distribution of scalp acupoints, we integrated our findings to determine the locations for clinical application.

### Literature screening and coordinate information organization

#### Data source and search strategy

Neurosynth Compose (https://compose.neurosynth.org) is a state-of-the-art platform providing a comprehensive database of brain imaging research and meta-analytic tools. While innovative, it has already demonstrated its effectiveness in numerous studies [10–12]. For this review, a combination of automated and manual analysis methods was employed to ensure a thorough and precise literature search. A broad query was conducted within the Neurosynth Compose database to identify all relevant studies on neuropsychiatric disorders published up to October 15, 2024. The search neurological diseases included Subjective Cognitive Decline (SCD), Mild Cognitive Impairment (MCI), Alzheimer’s Disease (AD), Parkinson’s Disease (PD), Multiple System Atrophy (MSA), Primary Progressive Aphasia (PPA), Post-Stroke Aphasia (PSA), Dyslexia, Chronic Pain, and Disorders of Consciousness (DoC).

#### Inclusion and exclusion criteria

Inclusion Criteria: (1) Studies involving participants diagnosed with the targeted neurological disorders; (2) Comparisons between patient groups and healthy controls, or between treatment and control groups for the targeted disorders; (3) Utilized neuroimaging modalities such as Magnetic Resonance Imaging (MRI), Positron Emission Tomography (PET), Single-Photon Emission Computed Tomography (SPECT), Arterial Spin Labeling (ASL), Electroencephalography (EEG), or Magnetoencephalography (MEG); (4) Reported 3D Talairach or MNI coordinates for between-group comparisons.

Exclusion Criteria: (1) Studies including only healthy participants and focusing on experimental models of neurological symptoms; (2) Research unrelated to the targeted neurological disorders, or where the disorder of interest is not the primary focus (e.g., secondary symptoms or comorbid conditions overshadowing the primary disorder); (3) Studies that do not provide standard space coordinates for analysis; (4) Neuroimaging studies conducted on non-human subjects; (5) Machine learning studies aimed at predicting treatment response rather than exploring neuroimaging findings; (6) Neuroimaging meta-analyses, reviews, or single case reports.

#### Data transparency

A persistent identifier was assigned to the dataset to ensure transparency and facilitate future research accessibility. The finalized dataset, along with detailed inclusion and exclusion parameters, is available for review in Table 1.

**Table 1:** Screening of Literature on Neurological Diseases

Disease	Retrieved Studies	Excluded Studies	Included Studies	Number of Analyses*	coordinates	Data Identifier and View
SCD	77	23	54	78	836	https://neurovault.org/collections/18684/
MCI	563	145	418	700	9664	https://identifiers.org/neurovault.collection:18437
AD	925	563	362	572	7345	https://identifiers.org/neurovault.collection:18451
PD	616	73	543	930	14,388	https://identifiers.org/neurovault.collection:18433
MSA	48	20	28	59	769	https://identifiers.org/neurovault.collection:18468
PPA	76	23	53	78	1373	https://identifiers.org/neurovault.collection:18453
PSA	88	5	83	128	2808	https://identifiers.org/neurovault.collection:18424
Dys	162	27	135	241	3942	https://identifiers.org/neurovault.collection:18431
CP	378	127	251	455	6859	https://identifiers.org/neurovault.collection:18469
DoC	119	94	25	34	498	https://identifiers.org/neurovault.collection:18663

*An analysis represents a single statistical contrast between any number of groups/conditions. *SCD* Subjective Cognitive Decline, *MCI* Mild Cognitive Impairment; AD Alzheimer’s Disease; *PD* Parkinson’s Disease; *MSA* Multiple System Atrophy; *PPA* Primary Progressive Aphasia; *PSA* Post-Stroke Aphasia; *Dys* Dyslexia, *CP* Chronic Pain; *DoC* Disorders of Consciousness

### Meta-analyses methodology

Neurosynth Compose was applied for data analysis. Coordinate-based meta-analysis was performed using MKDAChi2, based on the algorithm in Neurosynth Compose supported by NiMARE. The false discovery rate (FDR) correction with a 0.05 criterion was applied to adjust the t-values in the uniformity test map for each disease.

### Identifying brain targets from neuroimaging meta-analysis

**Fig. 1:**
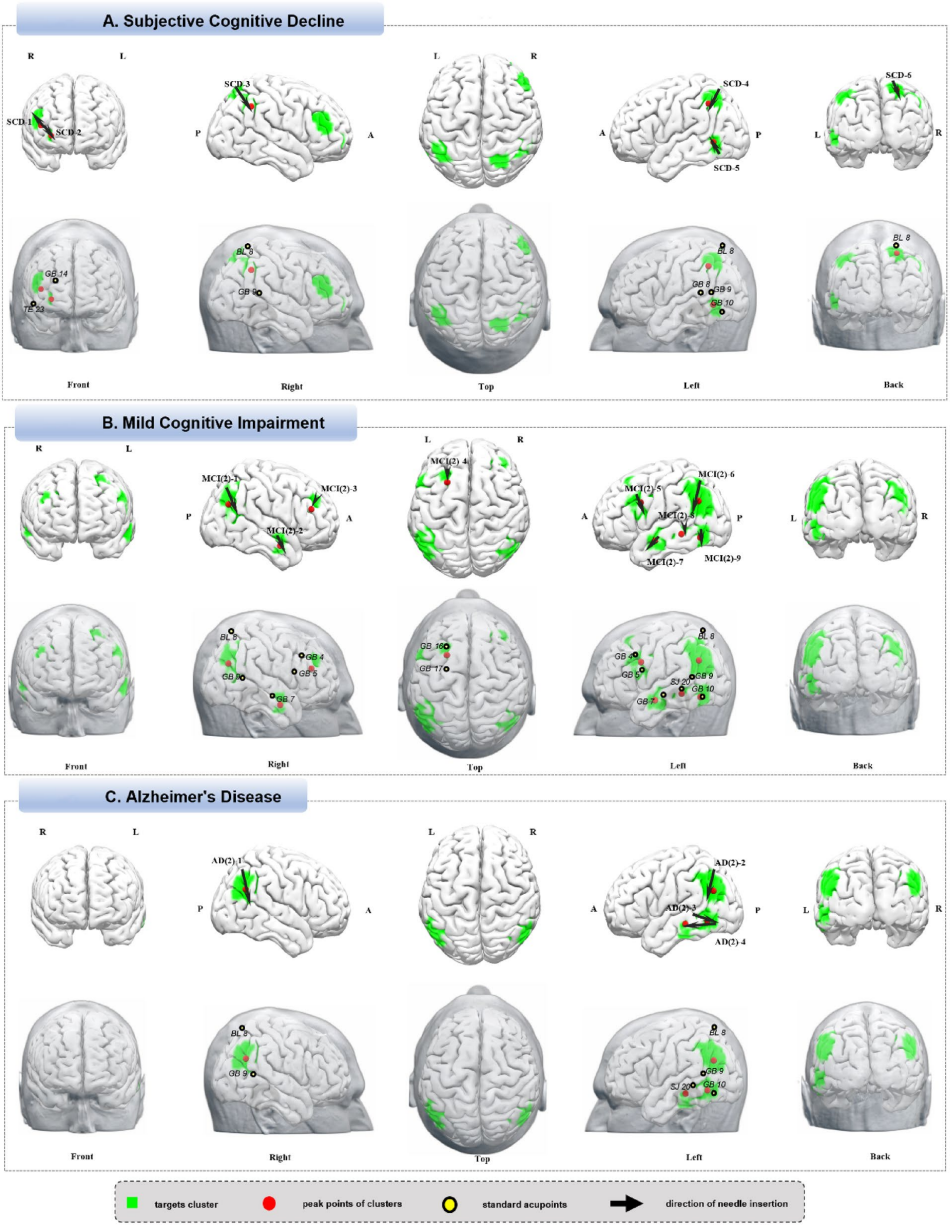
**A** Subjective cognitive decline. **B** Mild cognitive impairment. **C** Alzheimer’s Disease

The neuromodulation intervention methods, such as scalp acupuncture and transcranial electrical stimulation, primarily involve targeting the surface cerebral cortex. In light of this, a standard cortical brain template (within 2.5 cm of the scalp) [13] was applied to the uniformity test map to identify cortically accessible brain areas. Then, we used DPABI version 8.1 (http://rfmri.org/dpabi) to increase the T values in increments of 0.5 based on the uniformity test map, continuing this adjustment until 3 to 9 clusters are identified with voxel counts exceeding 30 and remaining under approximately 800 across all clusters, consistent with the approach used in our previous studies [8, 9]. For clusters extended different brain areas, we have provided separate peak coordinates, each representing different brain regions. The peak MNI coordinates of these clusters were reported using the xjView toolbox (http://www.alivelearn.net/xjview/) (AAL3 template[14]). The results were then mapped onto a standard brain using Surf Ice (www.nitrc.org/projects/surfice/) and a standard head using MRIcroGL (www.mccauslandcenter.sc.edu/mricrogl/), aligning with the international standard scalp acupuncture lines and acupoints (Supplementary Figs. 1A and Figs. 1B). We also provided the EEG standard 10–20 International System of Electrode Placement as an alternative localization method (Supplementary Figs. 1C).

### Identification of acupoints and needle application strategies

Following the Proposed Standard International Acupuncture Nomenclature published by the World Health Organization (WHO) in 1991, we confirmed the corresponding acupoints using the identified peak points. Furthermore, based on the three-dimensional spatial structure of the target brain cluster, we determined the needle direction and application strategies to maximize stimulation of the entire cluster. The process was completed collaboratively by two experienced acupuncturists.

## Results

We identified stimulation targets for 10 neurological disorders, with suggestion for each target’s needling direction, depth, and clinical application detailed in Tables 2–11. Supplementary Tables 1–10 provide additional targets, not derived from the surface cortical template, which encompass deeper brain regions that may be used by deep brain stimulation methods. To assist practitioners worldwide, Supplementary Table 11 provides the international standard names, along with the Chinese, Japanese, and Korean nomenclature for the acupoints. To differentiate the findings of this study from our previous research, we have added the notation “(2)” after targets identified for disorders that have been previously studied [8, 9].

**Table 2:** Neuroimaging-based targets for cranial acupuncture in subjective cognitive decline

Targets	Cluster size	T value	Peak MNI			Positioning and operation suggestions	Corresponding brain area
			X	Y	Z		
SCD-1	236	4.25	46	38	12	Horizontal insertion upward along the midpoint of the line between GB14 and TE23	R Inferior Frontal gyrus/ Middle Frontal Gyrus (PFC including DLPFC)
SCD-2	35	3.41	32	54	− 2	Horizontal insertion downward along the midpoint of the line between GB14 and TE23	R Middle/Superior Frontal Gyrus (PFC including DLPFC)
SCD-3	81	3.41	48	− 50	42	Horizontal insertion 0.5 cm posterior to BL8 toward GB9	R Inferior Parietal Lobule
SCD-4	193	6.42	− 42	− 52	46	Upper half of the line from BL8 to GB8	L Inferior Parietal Lobule/Angular Gyrus
SCD-5	41	4.25	− 52	− 58	− 2	From GB10 to GB9	L Middle/Inferior Temporal Gyrus
SCD-6	109	4.25	24	− 62	62	Horizontal insertion posteroinferiorly at BL8	R Superior Parietal Lobule

*MNI* Montreal Neurological Institute, *L* left, *R* right, *PFC* Prefrontal Cortex, *DLPFC* Dorsolateral Prefrontal Cortex

Scalp acupuncture follows a standardized procedural framework based on the Chinese National Standard (GB/T 33416–2016: *Specification of Manipulations of Acupuncture and Moxibustion—General Rules for the Drafting*) and the *World Federation of Acupuncture-Moxibustion Societies (WFAS) Technical Benchmark of Acupuncture and Moxibustion: Scalp Acupuncture* [15]. These protocols ensure safety, precision, and therapeutic efficacy by encompassing preoperative preparation, needle insertion, manipulation techniques, and post-treatment care. The integration of scalp acupuncture and non-invasive brain stimulation (NIBS) offers a structured approach for neuromodulation in neurological disorders.

In scalp acupuncture, preoperative preparation includes selecting single-use needles (0.25 mm or 0.30 mm diameter, 40 mm or 50 mm length, ISO 17218 compliant) to minimize trauma and bleeding. Precise acupoint localization is crucial and should align with the targeted cortical areas / line for specific neurological disorders (Tables 2–11). Needle insertion is performed at a 15–30° angle parallel to the scalp, reaching a depth of 1–3 cm into the subcutaneous layer of the galea aponeurotica, adjusted as needed. Stimulation is enhanced through twisting at 200 rotations per minute for 2–3 min, and electroacupuncture may be applied per *GB/T 21709.11 standards*. Post-treatment care involves gradual needle withdrawal, immediate site compression, and careful inspection of densely haired areas to prevent retained needles. Users may also modify needle insertion and manipulation techniques according to their personal clinical expertise or based on the suggestions of other specialists in the field.

### Subjective cognitive decline

Using search terms “Subjective Cognitive Decline,” “SCD,” “Subjective Cognitive Impairment,” “Subjective Memory Complaint,” and “Self-Reported Cognitive Decline,” 77 studies were initially retrieved. Twenty-three studies were excluded for lacking relevance or failing to separately identify SCD patients based on the exclusion criteria, resulting in 54 studies with 78 analyses and 836 brain coordinates (https://neurovault.org/collections/18684/).

Six scalp acupuncture targets were identified based on neuroimaging findings: SCD-1, the right inferior frontal gyrus and middle frontal gyrus; SCD-2, the right middle and superior frontal gyri; SCD-3, the right inferior parietal lobule; SCD-4, the left inferior parietal lobule and angular gyrus; SCD-5, the left middle and inferior temporal gyri; and SCD-6, the right superior parietal lobule (Fig. 1A, Table 2).

### Mild Cognitive Impairment

The search terms included “Mild Cognitive Impairment”, “Early Cognitive Decline”, “Pre-Dementia”, and “Mild Neurocognitive Disorder”. Of 563 indentified studies, 418 met criteria, with 700 analyses and 9,664 brain coordinates (https://identifiers.org/neurovault.collection:18437). The neuroimaging findings identified nine acupuncture targets: MCI (2)-1, the right middle occipital gyrus, angular gyrus, parietal lobule, and superior temporal gyrus; MCI (2)-2, the right middle temporal gyrus (MTG); MCI (2)-3, the right middle and superior frontal gyri (DLPFC); MCI (2)-4, the left superior and middle frontal gyri (dorsolateral prefrontal cortex, DLPFC); MCI (2)-5, the precentral gyrus and inferior frontal gyrus (IFG); MCI (2)-6, the angular gyrus, supramarginal gyrus, and superior parietal lobule; MCI (2)-7, the left MTG; MCI (2)-8, the left MTG; and MCI (2)-9, the left inferior occipital gyrus, middle occipital gyrus, and inferior temporal gyrus (Fig. 1B, Table 3).

**Table 3:** Neuroimaging-based targets for cranial acupuncture in mild cognitive impairment

Targets	Cluster size	T value	Peak MNI			Positioning and operation suggestions	Corresponding brain area
			X	Y	Z		
MCI(2)-1	403	6.17	40	− 68	28	Posterior-inferior from BL8 to GB9(R)	R Middle Occipital Gyrus/ Angular Gyrus/ Parietal Lobule / Superior Temporal Gyrus
MCI(2)-2	30	5.11	56	− 2	− 22	GB7 anterior-inferior transverse insertion(R)	R Middle Temporal Gyrus
MCI(2)-3	183	5.64	36	38	22	1 cm anterior–superior to the line from GB4 to GB5(R)	R Middle/ Superior Frontal Gyrus (PFC including DLPFC)
MCI(2)-4	112	5.37	− 24	18	52	GB16 to GB17(L)	L Superior/Middle Frontal Gyrus (PFC including DLPFC)
MCI(2)-5	335	9.05	− 46	12	30	GB4 to GB5(L)	L Precentral gyrus/ Inferior Frontal Gyrus
MCI(2)-6	684	6.44	− 48	− 62	32	BL8 to GB9(L)	L Angular Gyrus /Supramarginal Gyrus/ Superior Parietal Lobule
MCI(2)-7	51	5.90	− 56	− 6	− 16	GB7 anterior-inferior transverse insertion(L)	L Middle Temporal Gyrus
MCI(2)-8	33	4.58	− 56	− 40	− 8	SJ20 inferior transverse insertion(L)	L Middle Temporal Gyrus
MCI(2)-9	85	5.37	− 48	− 64	− 12	1 cm superior to GB10, inferior transverse insertion(L)	L Inferior and Middle Occipital Gyrus/ Inferior Temporal Gyrus

*MNI* Montreal Neurological Institute, *L* left, *R* right, *PFC* Prefrontal Cortex, *DLPFC* Dorsolateral Prefrontal Cortex

### Alzheimer’s Disease

The search terms included “Alzheimer’s Disease,” “AD”, “Senile Dementia”, and “Neurocognitive Disorder due to Alzheimer’s Disease”. After exclusions, 362 studies remained, yielding 572 analyses and 7,345 brain coordinates (https://identifiers.org/neurovault.collection:18451). Neuroimaging findings identified four acupuncture targets: AD (2)-1, the right angular gyrus and supramarginal gyrus; AD (2)-2, the left angular gyrus and supramarginal gyrus; AD (2)-3, the left  middle and inferior temporal gyri; and AD (2)-4, the left inferior temporal gyrus and occipitotemporal gyrus (Fig. 1C, Table 4).

**Table 4:** Neuroimaging-based targets for cranial acupuncture in Alzheimer’s disease

Targets	Cluster size	T value	Peak MNI			Location and operation suggestions	Corresponding brain area
			X	Y	Z		
AD(2)-1	466	6.92	48	− 60	30	BL8 to GB9(R)	R Angular Gyrus/ Supramarginal Gyrus
AD(2)-2	592	8.39	− 48	− 66	28	BL8 to GB9 (L)	L Angular gyrus/ Supramarginal Gyrus
AD(2)-3	83	5.14	− 58	− 30	− 12	GB10 anterior transverse insertion(L)	L Middle/ Inferior Temporal Gyrus
AD(2)-4	121	4.84	− 52	− 58	− 8	SJ20 to GB10(L)	L Inferior Temporal Gyrus/ Occipitotemporal Gyrus

*MNI* Montreal Neurological Institute, *L* left, *R* right

### Parkinson’s disease

**Fig. 2:**
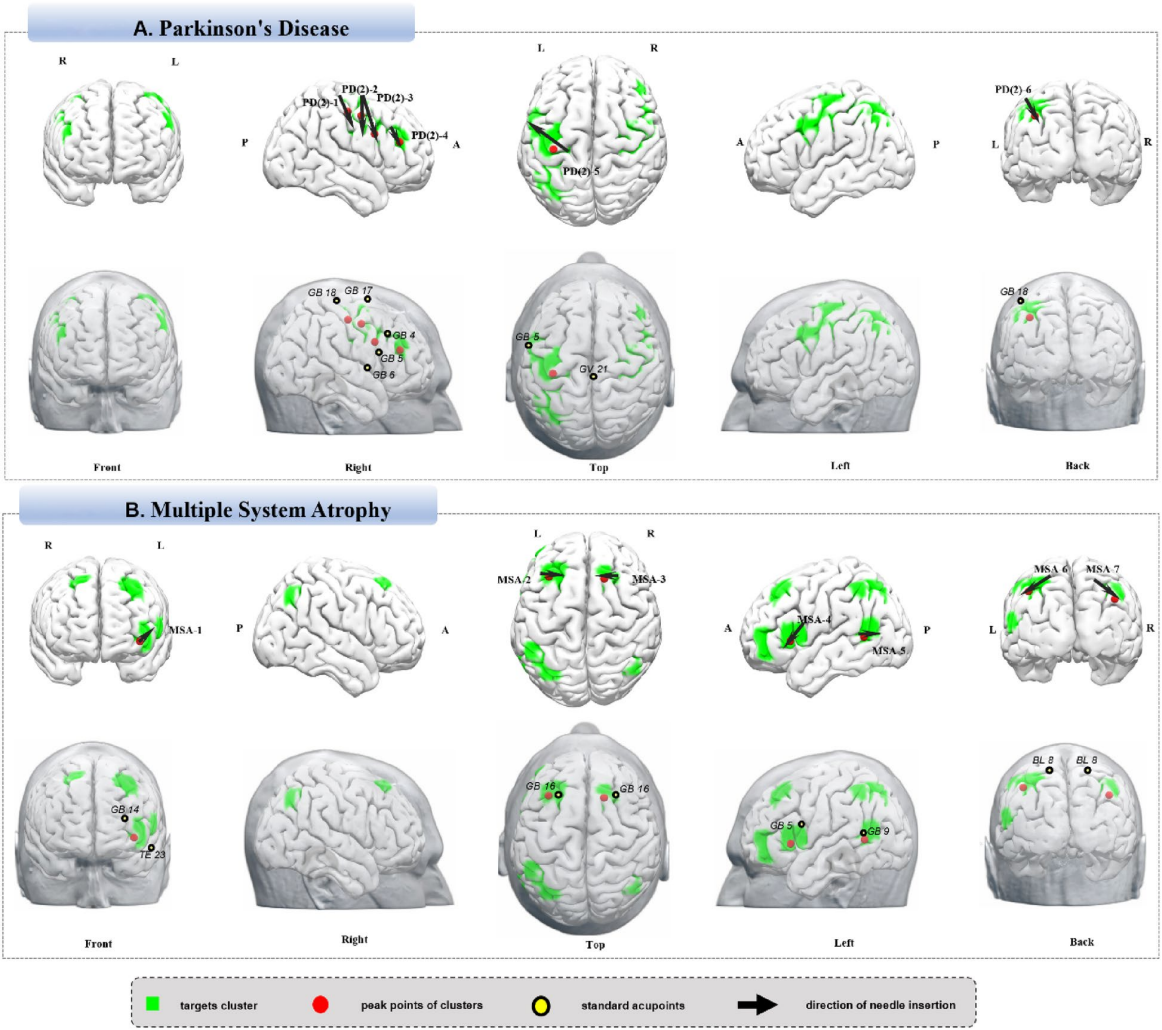
**A** Parkinson’s disease. **B** Multiple system atrophy

For search terms “Parkinson’s Disease”, “PD”, “Idiopathic Parkinsonism”, and “Primary Parkinsonism”, 543 studies met criteria, with 930 analyses and 14,388 brain coordinates (https://identifiers.org/neurovault.collection:18433). Neuroimaging findings identified six acupuncture targets: PD (2)-1, the right precentral gyrus and postcentral gyrus; PD (2)-2, the right precentral gyrus and middle frontal gyrus; PD (2)-3, the right IFG, middle frontal gyrus, and precentral gyrus; PD (2)-4, the right middle frontal gyrus (DLPFC); PD (2)-5, the left precentral gyrus, postcentral gyrus, middle frontal gyrus, and IFG; and PD (2)-6, the left angular gyrus and inferior parietal lobule (Fig. 2A, Table 5).

**Table 5:** Neuroimaging-based targets for cranial acupuncture in Parkinson’s disease

Targets	Cluster size	T value	Peak MNI			Location and operation suggestions	Corresponding brain area
			X	Y	Z		
PD(2)-1	45	5.43	40	− 16	52	Upper 2/3 of the line from GB18 to GB6(R)	R Precentral Gyrus/ Postcentral Gyrus
PD(2)-2	57	5.65	38	− 2	48	Upper 2/3 of the line from GB17 to GB6(R)	R Precentral Gyrus/ Middle Frontal Gyrus
PD(2)-3	250	6.77	48	12	30	GB17 to GB5(R)	R Inferior Frontal Gyrus/ Middle Frontal Gyrus/ Precentral Gyrus
PD(2)-4	158	6.10	38	38	22	GB4 anterior-inferior oblique insertion, 1–2 cm(R)	R Frontal Middle Gyrus (PFC including DLPFC)
PD(2)-5	697	9.16	− 36	− 24	56	Lower 2/3 of the line from GV21 to GB5(L)	L Precentral Gyrus/ Postcentral Gyrus / Middle Frontal Gyrus/ Inferior Frontal Gyrus
PD(2)-6	195	6.32	− 36	− 62	44	GB18 posterior insertion, 1–2 cm(L)	L Angular Gyrus/ Inferior Parietal Lobule

*MNI* Montreal Neurological Institute, *L* left, *R* right, *PFC* Prefrontal Cortex, *DLPFC* Dorsolateral Prefrontal Cortex

### Multiple system atrophy

Using terms “Multiple System Atrophy”, “Multiple System Atrophies”, “Multisystem Atrophy” and “Multiple System Atrophy Syndrome”, 28 studies were selected, resulting in 59 analyses and 769 coordinates (https://identifiers.org/neurovault.collection:18468). Neuroimaging findings identified seven targets: MSA-1, the left middle frontal gyrus (DLPFC) and IFG; MSA-2, the left middle and superior frontal gyri (DLPFC); MSA-3, the right superior frontal gyrus (DLPFC); MSA-4, the left IFG; MSA-5, the left MTG; MSA-6, the left inferior and superior parietal gyri; and MSA-7, the right angular gyrus and superior parietal lobule (Fig. 2B, Table 6).

**Table 6:** Neuroimaging-based targets for cranial acupuncture in multiple system atrophy

Targets	Cluster size	T value	Peak MNI			Location and operation suggestions	Corresponding brain area
			X	Y	Z		
MSA-1	124	2.22	− 38	46	− 6	Midpoint of the line from GB14 to TE23, posterior transverse insertion(L)	L Middle Frontal Gyrus (PFC including DLPFC)/ Inferior Frontal Gyrus
MSA-2	349	3.10	− 34	28	38	2 cm lateral to GB16, medial transverse insertion(L)	L Middle/Superior Frontal Gyrus (LPFC including DLPFC)
MSA-3	64	2.22	18	26	50	GB16 medial transverse insertion(R)	R Superior Frontal Gyrus/ (PFC including DLPFC)
MSA-4	189	2.22	− 50	22	2	GB5 anterior-inferior transverse insertion(L)	L Inferior Frontal Gyrus
MSA-5	88	2.22	− 52	− 54	6	GB9 posterior transverse insertion(L)	L Middle Temporal Gyrus
MSA-6	238	2.22	− 42	− 56	46	BL8 posterior-lateral transverse insertion (L)	L Inferior/Superior Parietal Gyrus
MSA-7	67	1.29	44	− 62	38	BL8 posterior-lateral transverse insertion (R)	R Angular Gyrus/ Superior Parietal Lobule

*MNI* Montreal Neurological Institute, *L* left, *R* right, *PFC* Prefrontal Cortex, *DLPFC* Dorsolateral Prefrontal Cortex

### Primary Progressive Aphasia

**Fig. 3:**
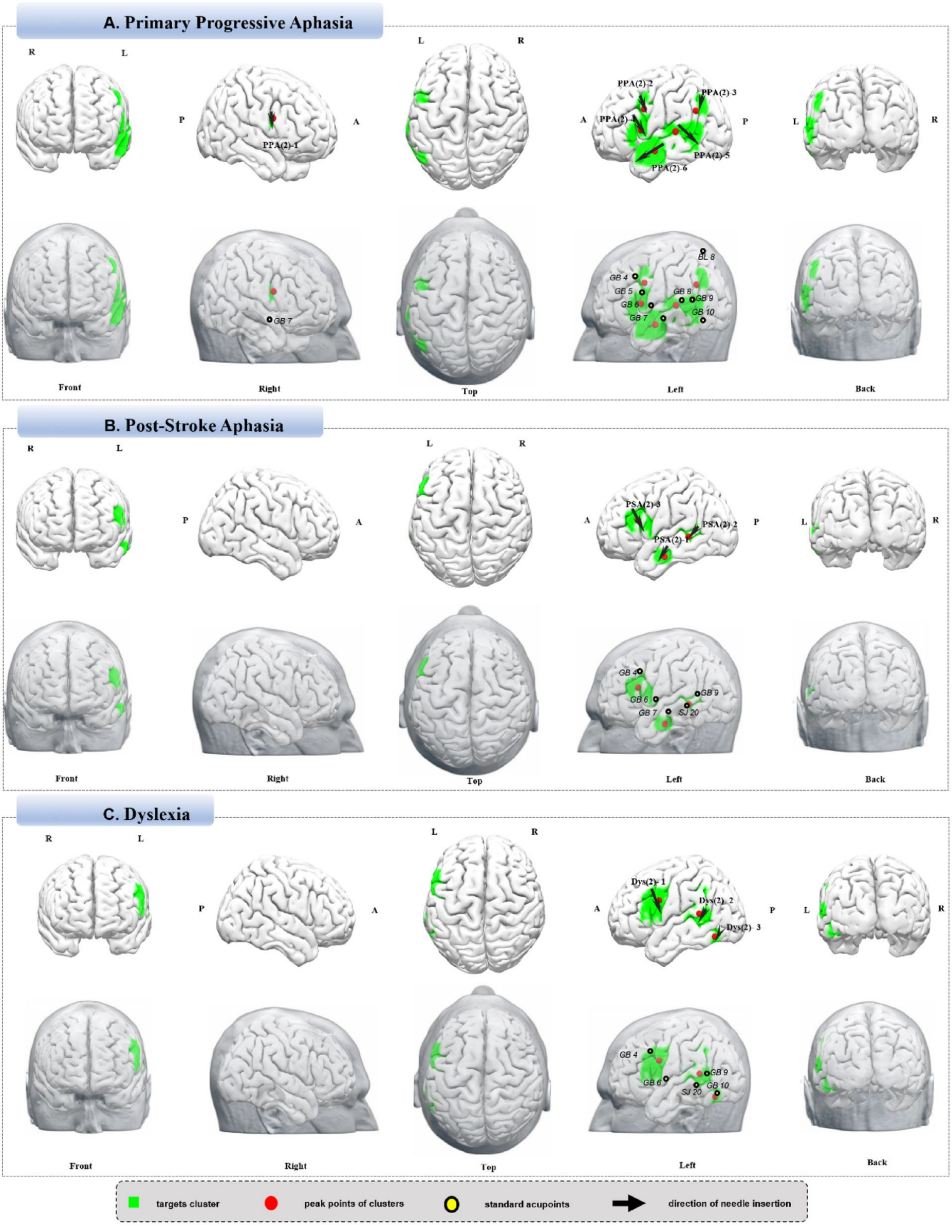
**A** Primary progressive aphasia. **B** Post-stroke aphasia. **C** Dyslexia

With “Primary Progressive Aphasia”, “Primary Aphasia”, “Aphasia”, “Language Disorder”, and “Progressive Nonfluent Aphasia”, 53 studies were selected, totaling 78 analyses and 1,373 coordinates (https://identifiers.org/neurovault.collection:18453). Neuroimaging findings identified six targets: PPA (2)-1, the right IFG; PPA (2)-2, the left precentral gyrus and middle frontal gyrus; PPA (2)-3, the left angular gyrus and inferior parietal lobule; PPA (2)-4, the left IFG; PPA (2)-5, the left temporal gyrus; and PPA (2)-6, the left temporal gyrus (Fig. 3A, Table 7).

**Table 7:** Neuroimaging-based targets for cranial acupuncture in primary progressive aphasia

Targets	Cluster size	T value	Peak MNI			Location and operation suggestions	Corresponding brain area
			X	Y	Z		
PPA(2)-1	39	4.92	52	14	20	3 cm superior to GB7, inferior insertion, 1–2 cm(R)	R Inferior Frontal Gyrus
PPA(2)-2	104	4.92	− 46	8	32	0.5 cm posterior to the line from GB4 to GB5(L)	L Precentral Gyrus/Middle Frontal Gyrus
PPA(2)-3	52	4.27	− 48	− 58	30	Middle third of the line from BL8 to GB9(L)	L Angular Gyrus/ Inferior Parietal Lobule
PPA(2)-4	165	5.58	− 52	12	8	1 cm anterior to the line from GB5 to GB6(L)	L Inferior Frontal Gyrus
PPA(2)-5	528	8.17	− 56	− 32	6	GB8 to GB10(L)	L Temporal Gyrus
PPA(2)-6	285	8.17	− 56	− 6	− 16	GB7 anterior-inferior transverse insertion(L)	L Temporal Gyrus

*MNI* Montreal Neurological Institute, *L* left, *R* right

### Post-Stroke Aphasia

Eighty-three studies provided 128 analyses and 2,808 coordinates for “Stroke Aphasia”, “Stroke Dysphasia” or “Stroke Language” (https://identifiers.org/neurovault.collection:18424). Neuroimaging findings identified three targets: PSA (2)-1, the left MTG; PSA (2)-2, the left middle and superior temporal gyri; and PSA (2)-3, the left inferior and middle frontal gyri (DLPFC) (Fig. 3B, Table 8).

**Table 8:** Neuroimaging-based targets for cranial acupuncture in post-stroke aphasia

Targets	Cluster size	T value	Peak MNI			Location and operation suggestions	Corresponding brain area
			X	Y	Z		
PSA(2)-1	43	7.50	− 58	− 12	− 24	GB7 anterior-inferior transverse insertion(L)	L Middle Temporal Gyrus
PSA(2)-2	63	7.01	− 56	− 42	0	GB9 to SJ20(L)	L Middle/Superior Temporal Gyrus
PSA(2)-3	337	9.34	− 46	22	20	0.5 cm anterior to the line from GB4 to GB6(L)	L Inferior/Middle Frontal Gyrus (PFC including DLPFC)

*MNI* Montreal Neurological Institute, *L* left, *R* right, *PFC* Prefrontal Cortex, *DLPFC* Dorsolateral Prefrontal Cortex

### Dyslexia

Using the search terms “Dyslexia,” “Developmental Reading Disorder,” and “Reading Disability,” 162 studies were identified. From which 135 were included, yielding 241 analyses and 3,942 coordinates. (https://identifiers.org/neurovault.collection:18431). Neuroimaging findings identified three targets: Dys (2)-1, the left inferior and middle frontal gyri; Dys (2)-2, the left superior temporal gyrus, MTG, and insula; and Dys (2)-3, the left inferior occipital gyrus and inferior temporal gyrus (Fig. 3C, Table 9).

**Table 9:** Neuroimaging-based targets for cranial acupuncture in dyslexia

Targets	Cluster size	T value	Peak MNI			Location and operation suggestions	Corresponding brain area
			X	Y	Z		
Dys(2)-1	655	15.75	− 46	8	28	GB4 to GB6(L)	L Inferior/Middle Frontal Gyrus (PFC including DLPFC)
Dys(2)-2	273	9.99	− 56	− 44	12	GB9 to SJ20(L)	L Superior Temporal Gyrus /Middle Temporal Gyrus/ Insula
Dys(2)-3	39	9.57	− 46	− 64	− 16	1 cm superior to GB10, anterior-inferior transverse insertion(L)	L Inferior Occipital Gyrus/ Inferior Temporal Gyrus

*MNI* Montreal Neurological Institute, *L* left, *R* right, *PFC* Prefrontal Cortex, *DLPFC* Dorsolateral Prefrontal Cortex

### Chronic pain

**Fig. 4:**
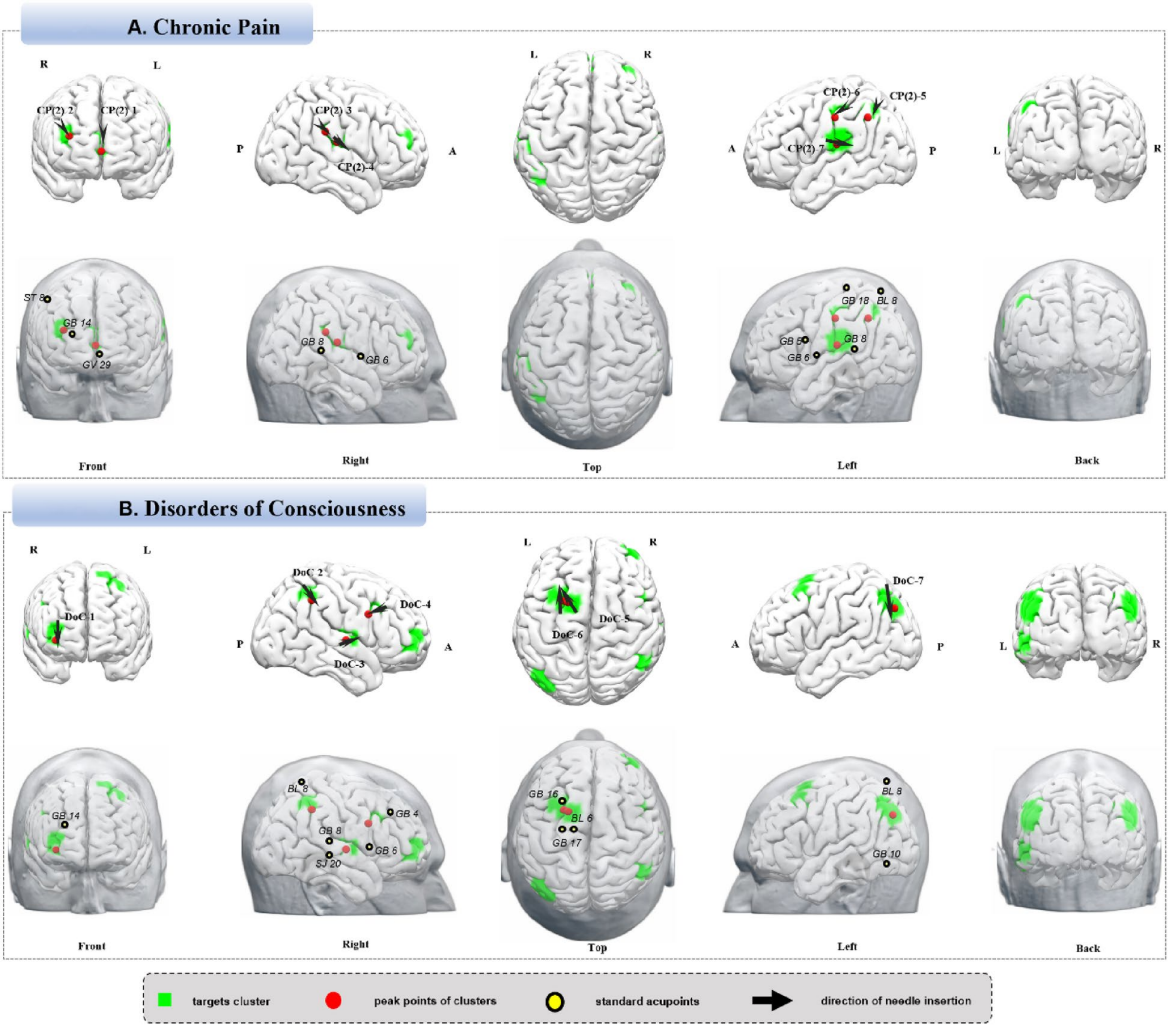
**A** Chronic pain. **B** Disorders of consciousness

Using terms “Chronic Pain”, “Widespread Chronic Pain”, “Persistent Pain”, and “Constant Pain”, 251 studies with 455 analyses and 6,859 coordinates were included for chronic pain (https://identifiers.org/neurovault.collection:18469). Neuroimaging findings identified seven targets: CP (2)-1, the right superior frontal gyrus ); CP (2)-2, the right superior frontal gyrus (DLPFC); CP (2)-3, the right supramarginal gyrus; CP (2)-4, the right rolandic opercular gyrus; CP (2)-5, the left inferior parietal lobule; CP (2)-6, the left precentral gyrus and postcentral gyrus (primary motor cortex and primary somatosensory cortex); and CP (2)-7, the left supramarginal gyrus, postcentral gyrus, and superior temporal gyrus (Fig. 4A, Table 10).

**Table 10:** Neuroimaging-based targets for cranial acupuncture in chronic pain

Targets	Cluster size	T value	Peak MNI			Location and operation suggestions	Corresponding brain area
			X	Y	Z		
CP(2)-1	137	5.72	2	58	2	1 cm superior to GV29, inferior transverse insertion(R)	R Superior Frontal Gyrus
CP(2)-2	45	4.48	34	48	18	Lower 1/3 of the line from ST8 to GB14(R)	R Superior Frontal Gyrus (PFC including DLPFC)
CP(2)-3	69	4.48	56	-32	28	2 cm superior to GB8, upper 1/3 of the line to GB6(R)	R SupraMarginal Gyrus
CP(2)-4	38	4.48	58	-20	18	2 cm superior to GB8, lower 2/3 of the line to GB6(R)	R Rolandic Opercular Gyrus
CP(2)-5	63	5.10	-44	-54	42	Upper 1/3 of the line from BL8 to GB8(L)	L Inferior Parietal Lobule
CP(2)-6	47	4.79	-48	-20	42	0.5 cm inferior to GB18, upper 1/3 of the line to GB5(L)	L Precentral Gyrus/ Postcentral Gyrus (Primary Motor Cortex/ Primary Somatosensory Cortex)
CP(2)-7	172	6.64	-58	-22	16	Posterior 1/2 of the line from GB5 to GB8(L)	L SupraMarginal Gyrus/ Postcentral Gyrus/ Superior Temporal Gyrus

*MNI* Montreal Neurological Institute, *L* left, *R* right, *PFC* Prefrontal Cortex, *DLPFC* Dorsolateral Prefrontal Cortex

### Disorders of consciousness

For “Consciousness Disorder”, “Disorder of Consciousness”, “Consciousness, Level Depressed”, “Depressed Level of Consciousness”, “Consciousness, Level Altered”, “Altered Level of Consciousness”, “Semiconsciousness”, “Unconsciousness” or “Coma”, “Unresponsive Wakefulness Syndrome” or “Minimally Conscious State”, 25 studies were included, totaling 34 analyses and 498 coordinates (https://identifiers.org/neurovault.collection:18663). Neuroimaging findings identified seven targets: DoC-1, the right middle frontal gyrus (DLPFC); DoC-2, the right supramarginal gyrus; DoC-3, the right superior temporal gyrus and rolandic opercular gyrus; DoC-4, the right IFG; DoC-5, the left superior frontal gyrus (DLPFC); DoC-6, the left superior and middle frontal gyri (DLPFC); and DoC-7, the left middle occipital gyrus, angular gyrus, and superior parietal lobule (Fig. 4B, Table 11).

**Table 11:** Neuroimaging-based targets for cranial acupuncture in disorders of consciousness

Targets	Cluster size	T value	Peak MNI			Location and operation suggestions	Corresponding brain area
			X	Y	Z		
DoC-1	57	2.10	36	54	− 4	Horizontal insertion downward at GB14	R Middle Frontal Gyrus (PFC including DLPFC)
DoC-2	69	3.09	48	− 44	44	Upper third along the line from BL8 to GB8	R SupraMarginal Gyrus
DoC-3	51	2.10	60	− 10	4	Horizontal insertion 1 cm anterior to SJ20 toward GB6	R Superior Temporal Gyrus/ Rolandic Opercular Gyrus
DoC-4	30	2.10	48	12	30	Posteroinferior horizontal insertion at a 45° angle at GB4	R Inferior Frontal Gyrus
DoC-5	52	2.10	− 20	14	60	From GB17 to GB16	L Superior Frontal Gyrus (PFC including DLPFC)
DoC-6	134	4.16	− 24	14	52	From BL6 to GB16	L Superior/Middle Frontal Gyrus (PFC including DLPFC)
DoC-7	318	4.16	− 38	− 76	38	Upper third along the line from BL8 to GB10	L Middle Occipital Gyrus/ Angular Gyrus/ Superior Parietal Lobule

*MNI* Montreal Neurological Institute, *L* left, *R* right, *PFC* Prefrontal Cortex, *DLPFC* Dorsolateral Prefrontal Cortex

## Discussion

Using Neurosynth Compose, this study identified scalp acupuncture stimulation targets for ten common neurological disorders. The identified regions/ networks for neurological disorders may also be used for non-invasive brain stimulation or mechanism studies of these disorders.

To explore the clinical feasibility of our new scalp acupuncture system, we have compared our findings with the protocols outlined in the latest *Standardized Manipulations of Acupuncture and Moxibustion of People’s Republic of China* (GB/T21709.2–2021) (*Standardized Manipulations*) and the *14th Five-Year Plan Textbook: Acupuncture and Moxibustion Therapy* (*Textbook*). Additionally, we compared our results with the widely recognized scalp acupuncture systems developed by Jiao Shunfa [16] and Jin Rui [17], known as Jiao’s Scalp Acupuncture and Jin’s Three-Needle Scalp Acupuncture System. While overlaps with these existing methods were observed, this study identified novel scalp stimulation targets that may supplement current practices and provide new therapeutic options for neurological disorders.

### Potential mechanisms underlying scalp acupuncture

Scalp acupuncture operates on the principle that stimulating specific scalp areas influences corresponding brain regions, leading to clinical benefits [18].

National and international organizations, including the WHO, have established standards based on this concept. Extensive clinical studies and decades of practice have demonstrated its efficacy, yet the precise mechanisms remain under investigation. Recent findings suggest a potential neural pathway—trigeminal nerve–meninges–cerebrospinal fluid–contacting neurons–brain—implicated in modulating brain function and treating cerebral diseases [18]. Experimental studies in migraine rat models indicate that scalp acupuncture exerts analgesic effects via the convergence of facial and dura mater inputs in wide dynamic range neurons [19]. Additionally, its impact on intracranial structures may involve presynaptic dorsal root reflexes, postsynaptic neurogenic responses, and convergent neural pathways[20].

In humans, research has revealed that stimulation along specific scalp acupuncture lines, such as MS5, MS6, and MS7, enhances functional connectivity in brain regions related to cognition, sensory integration, and motor coordination.[21].

Notably, electroacupuncture on the scalp is akin to transcranial electrical stimulation (tES), particularly alternating current stimulation (tACS), which can modulate neuronal oscillations and brain connectivity beyond the targeted area [22] [23] [24] [25]. Collectively, these findings demonstrated scalp acupuncture’s potential for brain disease treatment, validating its methodological applications in chronic pain and comorbid disorders [26].

### Identified targets for neurodegenerative diseases: SCD, MCI and AD

AD progresses through stages, beginning with SCD, followed by MCI, and culminating in dementia. SCD is characterized by self-reported cognitive decline without detectable deficits on standardized tests, whereas MCI represents a transitional phase with mild but measurable cognitive impairments. Both SCD and MCI significantly increase the risk of developing AD, highlighting their importance for early identification and intervention[27].

#### Neuroimaging insights and emerging scalp acupuncture targets

Neuroimaging studies suggest overlapping yet distinct brain alteration patterns across SCD, MCI and AD, emphasizing the need for tailored approaches in understanding and managing these stages[28]. Current scalp acupuncture protocols, including those outlined in the GB/T21709.2–2021 standard, recommend several key stimulation lines for cognitive decline and AD, such as MS1 (middle line of the forehead), MS5 (middle line of the vertex), MS10 (anterior temporal line), and MS11 (posterior temporal line). Additionally, Jin’s “four divine needles” and “three wisdom needles” are commonly used in clinical practice for cognitive disorders.

We identified several new scalp acupuncture points that may have clinical implication. While further clinical trials are necessary to evaluate their effectiveness, our findings are supported by studies using NIBS techniques to enhance cognitive functions for individuals with SCD, MCI and in early stages of AD [29–32]. The stimulation sites used in these studies, including the middle frontal gyrus (SCD-2, MCI (2)-3), MTG (SCD-5), and occipital gyrus (MCI (2)-1, MCI (2)-9), align closely with the targets identified in our research, supporting their clinical relevance.

#### Functional roles of key brain regions

Many clinical studies [33–37] have demonstrated the potentials of neuromodulation on brain regions such as the left angular gyrus, parietal lobule, and MTG in improving cognitive functions, including memory, learning, and active cognition. These findings align closely with our identified targets, including SCD-1, SCD-3, SCD-4, SCD-6, MCI (2)-6, and MCI (2)-8, further validating the clinical relevance of our results.

For example, the angular gyrus plays a central role in modulating memory and cognitive functions through its connectivity with the hippocampus and the default mode network (DMN) [38](MCI Group MoCA Effect Sizes: 0.672; AD Group MoCA Effect Sizes: 0.636). Similarly, the temporal gyrus has been implicated in mitigating early cognitive impairments by enhancing semantic processing and verbal fluency [39, 40].

Additionally, previous studies[41–43] has emphasized the roles of the bilateral DLPFC, temporal, and parietal lobes in improving memory, executive function, and emotional regulation in patients with AD. Meta-analysis demonstrated that NIBS combined with cognitive training effectively improved global cognition in AD and MCI (SMD = 0.52, 95% CI [0.18, 0.87], p = 0.003), particularly in patients receiving rTMS combined with cognitive training (SMD = 0.46, 95% CI [0.14, 0.78], p = 0.005). These findings are consistent with our identified targets in these regions and highlight their potential in addressing cognitive and emotional deficits associated with AD.

#### Stage-specific and shared neural patterns

Distinct regions highlight the unique characteristics of each condition. SCD is marked by early disruptions in executive function (e.g., right inferior frontal triangular gyrus) and visuospatial processing (e.g., right superior parietal lobule). MCI introduces additional involvement of occipital regions (e.g., right middle occipital gyrus) and frontal regions (e.g., left precentral gyrus), indicating emerging deficits in visual processing and motor function. AD is characterized by pronounced impairments in advanced memory and semantic processing, with significant disruptions in the inferior temporal gyrus. These findings provide a foundation for designing disease-specific therapeutic interventions, including neuroimaging-guided scalp acupuncture, to address both shared and unique neural deficits across the spectrum of cognitive decline.

#### Bridging surface and deep brain connectivity

The comparative analysis of brain regions associated with SCD, MCI, and AD highlights shared condition-specific neural disruptions. Parietal regions, particularly the angular gyrus, demonstrate consistent involvement across all three conditions, indicating their central role in spatial attention, multimodal integration, and memory [38] [44]. Temporal regions, including the middle temporal gyrus and inferior temporal gyrus, reflect a continuum of memory and language deficits as the condition progresses from SCD to AD [40].

While our scalp acupuncture cannot directly stimulate deep brain regions such as the hippocampus, an alternative approach from our previous study [45] targets the functional and anatomical connectivity of deep structures, including the hippocampus, through sites near GV19, is in alignment with traditional acupuncture protocols for dementia.

### Identified targets for movement disorders: Parkinson’s disease and multiple system atrophy

Movement disorders, including PD and MSA, are characterized by progressive motor and non-motor impairments resulting from complex neural disruptions affecting key motor and cognitive pathways. Symptoms range from tremors and rigidity to autonomic dysfunction and cerebellar ataxia [46]. A variety of NIBS techniques have been utilized to mitigate these impairments by targeting key cortical areas involved in motor control and coordination [47] [48]. Moderator variable analysis revealed that NIBS targeting the DLPFC significantly enhanced cognitive performance during dual-task conditions (SMD = 0.283, SE = 0.099, 95% CI = 0.089–0.478, Z = 2.860, P = 0.004, I^2^ = 20.9%), suggesting that tDCS applied to the DLPFC may contribute to small but meaningful improvements in cognitive performance during dual-task paradigms, with minimal heterogeneity.

This section examines current scalp acupuncture and NIBS approaches, emphasizing both established and novel targets identified in this study.

#### Parkinson’s disease

In traditional Chinese medicine, PD falls under the category of “tremor and spasm disease”. Jiao’s scalp acupuncture suggests targeting “dance tremors” scalp area, while Jin’s scalp acupuncture recommends three temporal needles (Niesan needles) and three brain needles (Naosan needles). Consistent with these established acupuncture protocols, our findings identified targets within the “dance tremors” area (e.g., PD (2)-3). Additionally, we have identified targets in the motor cortex and DLPFC regions (e.g., PD (2)-2/4).

The central mechanisms of PD predominantly involve degeneration of the nigrostriatal pathway. However, recent research has extended the focus to the basal ganglia-thalamo-cortical loop [49]. NIBS has emerged as a promising treatment modality for PD, targeting various cortical areas to address both motor and non-motor symptoms. Recent studies have shown that high-frequency rTMS at M1, DLPFC and the cerebellum can improve motor symptoms such as dyskinesia and depression. Similarly, anodal tDCS over M1 or combined M1 + DLPFC stimulation may improve motor symptoms such as freezing of gait [50, 51].

In alignment with these findings, we identified brain targets in regions including M1 and the DLPFC (e.g., PD (2)-1 to PD (2)-5). Furthermore, we also identified additional brain targets in the angular gyrus (PD (2)-6), which may be associated with the cognitive and motor symptoms of PD. Modulation of this area may provide a dual benefit, addressing both motor and non-motor symptoms for PD [52]. These findings highlight the need for further research to explore the therapeutic potential of targeting the angular gyrus in PD treatment.

#### Multiple system atrophy

The *Textbook* recommend the lower-lateral line of the occiput (MS14) for balance disorders caused by cerebellar dysfunction, making it a suitable target for MSA-C (cerebellar type). Similarly, Jiao’s scalp acupuncture suggests the “balance area,” which corresponds to MS14, as the main stimulation area for balance-related impairments.

Recent research highlights the potential of NIBS in managing motor symptoms and cerebellar dysfunction for MSA. A systematic review [48] showed that rTMS and tDCS are the most studied NIBS modalities for both MSA-P (parkinsonian type) and MSA-C. In MSA-P, the primary motor cortex was the most frequently targeted area, whereas in MSA-C, the cerebellum was the focus of stimulation. Given the role of the cerebello-thalamocortical circuit in motor control, targeting both the cerebellum and M1 is critical for treating motor impairments in MSA [53]. Emerging evidence, such as studies on cerebellar theta-burst stimulation (iTBS), has shown improvements in motor balance and cerebello-frontal connectivity, further emphasizing the cerebellum as a key therapeutic target [54].

In this study, we identified DLPFC (MSA-1/2/3), IFG, temporal and parietal gyrus MSA as potential targets. The DLPFC, crucial for executive functions and motor control, has shown potential in improving connectivity with motor and cognitive circuits, thereby enhancing cognition and motor performance. A previous study [55] have found that rTMS over the left DLPFC provided short-term alleviation of fatigue in MSA patients, with reductions in fatigue severity and some motor symptoms lasting up to two weeks before diminishing by four weeks. This suggests that the DLPFC may modulate both motor and non-motor symptoms in MSA, warranting further research on its potential for broader symptom relief.

The IFG, essential for response inhibition and motor planning, could be a promising target to improve motor coordination and inhibitory control deficits. The temporal gyrus, central to auditory processing, memory, and emotional regulation, offers potential for addressing memory impairments and emotional dysregulation for MSA. Finally, the parietal gyrus, which integrates sensory and motor information and supports visuospatial attention, may improve motor coordination and sensory-motor integration. Together, these regions provide a foundation for targeted NIBS interventions to manage both motor and non-motor symptoms of MSA, nevertheless further research is necessary to establish their efficacy.

### Identified targets for language and language-related disorders: post-stroke aphasia, primary progressive aphasia, and dyslexia

Language and reading disorders, including PSA, PPA, and dyslexia, are associated with disruptions in neural networks involved in language and reading functions. This section describes therapeutic approaches, and the stimulation targets identified in this study.

#### Post-stroke aphasia and primary progressive aphasia

The *Standardized Manipulations* recommends the lower two fifths of the anterior oblique line of the vertex-temporal (MS6) and the anterior temporal line (MS10) for treating motor aphasia. Similarly, Jiao’s scalp acupuncture suggests “verbal region 2” for anomia and “verbal area 3” for sensory aphasia, corresponding closely to the MS6 and MS10 lines. Our study further refined these scalps stimulation targets, providing more specific localization for therapeutic applications.

For post-stroke aphasia (PSA), techniques such as rTMS and tDCS have shown promise in enhancing language recovery by targeting specific brain areas. rTMS is typically used to reduce overactivity (effect size = 1.01) in the contra lesional hemisphere by inhibiting the right pars triangularis (part of the right IFG). Conversely, tDCS is often applied to the left perilesional Broca’s area (within the left IFG) to boost excitability and support neuroplasticity [56].

For primary progressive aphasia (PPA), applying anodal tDCS to the left DLPFC has helped improve naming and daily communication, particularly in the agrammatic variant of PPA [57]. Consistent with these findings and the critical role of the left MTG in language processing [58], we identified targets in the left IFG and MTG. These regions can be stimulated individually based on lesion location or bilaterally to engage both hemispheres, offering a comprehensive approach for PSA and PPA treatment. Further research is required to confirm the effectiveness of these interventions in promoting language recovery and improving communication.

#### Dyslexia

Neither The Standardized *Manipulations* nor the *Textbook* includes acupuncture recommendations for dyslexia. We have identified several targets for dyslexia.

The left IFG (Dys (2) -1) is responsible for speech production and language output; the left superior temporal gyrus (Dys (2) -2) supports speech perception and language comprehension; and the left inferior occipital gyrus (Dys (2) -3) is involved in text decoding and visual information processing. These three regions form the core components of the “reading neural network” and are closely associated with typical reading fluency skills[59, 60].

Studies on NIBS often target the temporoparietal junction, posterior superior and MTG, inferior parietal lobule, and IFG. Stimulating the temporoparietal junction has been shown to improve reading accuracy and speed, while targeting the posterior superior and middle temporal gyri can enhance reading speed. Modulating the IFG may improve speech perception, and stimulating the inferior parietal lobule can increase the accuracy and speed of non-word reading [61]. The targets identified in this study align closely with those in previous research on dyslexia, providing evidence to support the validity of the selected targets.

### Identified targets for chronic pain, and disorders of consciousness

#### Chronic pain

The *Standardized Manipulations* recommends different acupuncture therapy according to the locations chronic pain conditions. For instance, the middle line of vertex (MS5) is indicated for waist and leg pain, while line 1 lateral to the vertex (MS8) is used for waist, leg and foot pain, and the 2 lateral to the vertex (MS9) is applied for shoulders, arms, hands pain.

NIBS techniques have emerged as promising tools for chronic pain management, although their efficacy varies across studies. In a comprehensive literature review [7] on brain surface targets for NIBS in treating chronic pain disorders, we found that the primary motor cortex (M1; corresponding to C3/C4 in the 10–20 EEG system) and the lateral prefrontal cortex (F3/F4/Fz) are the most frequently targeted regions for chronic pain treatment. Consistent with these findings, our study identified similar targets but with more focused and precise localization, offering potential for enhanced therapeutic outcomes.

#### Disorders of consciousness

The *Standardized Manipulations* and related textbook do not specifically recommend scalp acupuncture therapies for DoC. Similarly, neither Jiao’s nor Jin’s scalp acupuncture protocols include explicit recommendations for this condition, highlighting a critical area for further investigation.

Literature suggest that DoC are associated with dysfunction in neural circuits such as the frontoparietal network, DMN, and salience network, with key regions including the DLPFC, thalamus, and precuneus. Recent advances in NIBS for DoC have demonstrated the potential to modulate consciousness levels by targeting these neural circuits. For instance, systematic reviews [62, 63] have showed that anodal tDCS over the left DLPFC significantly improves consciousness scores, particularly in individuals with minimally conscious state, by enhancing cortical excitability and functional connectivity within the frontoparietal network.

Consistent with these findings, our study has identified brain targets located in the DLPFC as potential intervention points for DoC. Furthermore, we have identified additional targets in the superior temporal gyrus, supramarginal gyrus, and middle occipital gyrus. The superior temporal gyrus plays a key role in auditory processing and language comprehension, making it a potential target for improving auditory awareness and communication in DoC[64]. The supramarginal gyrus is involved in sensory integration and spatial awareness, stimulating the area may enhance responsiveness to external stimuli [65] and coordination [66]. The middle occipital gyrus, which is critical for visual processing, may be targeted to improve visuospatial and perceptual awareness in these patients [67].

Targeting together, stimulating these regions through scalp acupuncture or NIBS may modulate cortical activity, enhance connectivity within disrupted neural networks, and improve awareness and responsiveness in individuals with DoC. Nevertheless, further research is needed to explore these targets’ therapeutic potential and optimize intervention strategies for this complex condition.

### Limitations

This study has several limitations. First, as it relies on the Neurosynth Compose platform, the identified cortical targets are derived from meta-analytic literature and may evolve as more high-quality studies emerge. While our findings differ slightly from previous research[7–9] due to improved data availability, future updates and individualized stimulation protocols are needed to refine target selection.

Second, while some identified targets align with prior brain stimulation and acupuncture research, others remain theoretical and require clinical validation. Our scalp projections do not account for deep brain structures, as we believe that scalp acupuncture cannot directly reach deeper brain structures. Future integration of functional connectivity and white matter tractography of deep structure to surface area may help bridge this gap [68]. Lastly, while we provide target selection and needling direction, specificity of the stimulation targets across different disease state, treatment parameters such as stimulation intensity, frequency, and duration remain beyond this study’s scope.

Despite these limitations, this study establishes a systematic, neuroimaging-guided framework for scalp acupuncture target identification, offering valuable insights for optimizing evidence-based interventions in neurological disorders.

## Conclusion

We have identified potential scalp acupuncture targets for ten neurological disorders. These targets can also be applied to brain stimulation methods. Identifying these targets / brain areas may advance the development of scalp acupuncture and brain stimulation as well as the brain imaging research for these disorders.

## Supplementary Information

Supplementary Material 1.

## Data Availability

The datasets generated and analyzed for this study are available in the Neurosynth Compose neuroimaging literature analysis platform repository. Links to the datasets and images for each disease mentioned in the article are provided in Table 1.

## Abbreviations

AD: Alzheimer’s Disease
ASL: Arterial Spin Labeling
BA: Brodmann Area
DLPFC: Dorsolateral Prefrontal Cortex
DMN: Default Mode Network
DoC: Disorders of Consciousness
EEG: Electroencephalography
FDR: False Discovery Rate
FPN: Frontoparietal Network
GV: Governing Vessel
IFG: Inferior Frontal Gyrus
M1: Primary Motor Cortex
MCI: Mild Cognitive Impairment
MEG: Magnetoencephalography
MNI: Montreal Neurological Institute
MRI: Magnetic Resonance Imaging
MSA: Multiple System Atrophy
MTG: Middle Temporal Gyrus
NIBS: Non-Invasive Brain Stimulation
PPA: Primary Progressive Aphasia
PSA: Post-Stroke Aphasia
rTMS: Repetitive Transcranial Magnetic Stimulation
SCD: Subjective Cognitive Decline
SPECT: Single-Photon Emission Computed Tomography
tDCS: Transcranial Direct Current Stimulation
TCM: Traditional Chinese Medicine
WFAS: World Federation of Acupuncture-Moxibustion Societies
WHO: World Health Organization
