# Supplementary material for: Potential scalp acupuncture and brain stimulation targets for common neurological disorders: evidence from neuroimaging studies
Source: Chin Med. 2025 May 7;20:58. doi: 10.1186/s13020-025-01106-0 (PMC12057072; doi:10.1186/s13020-025-01106-0)
Supplement: Supplementary file 1 — Supplementary Material 1. [file 13020_2025_1106_MOESM1_ESM.docx]

**Supplementary Figures 1 Pipeline for Identifying Scalp Acupuncture and Brain Stimulation Targets**

**
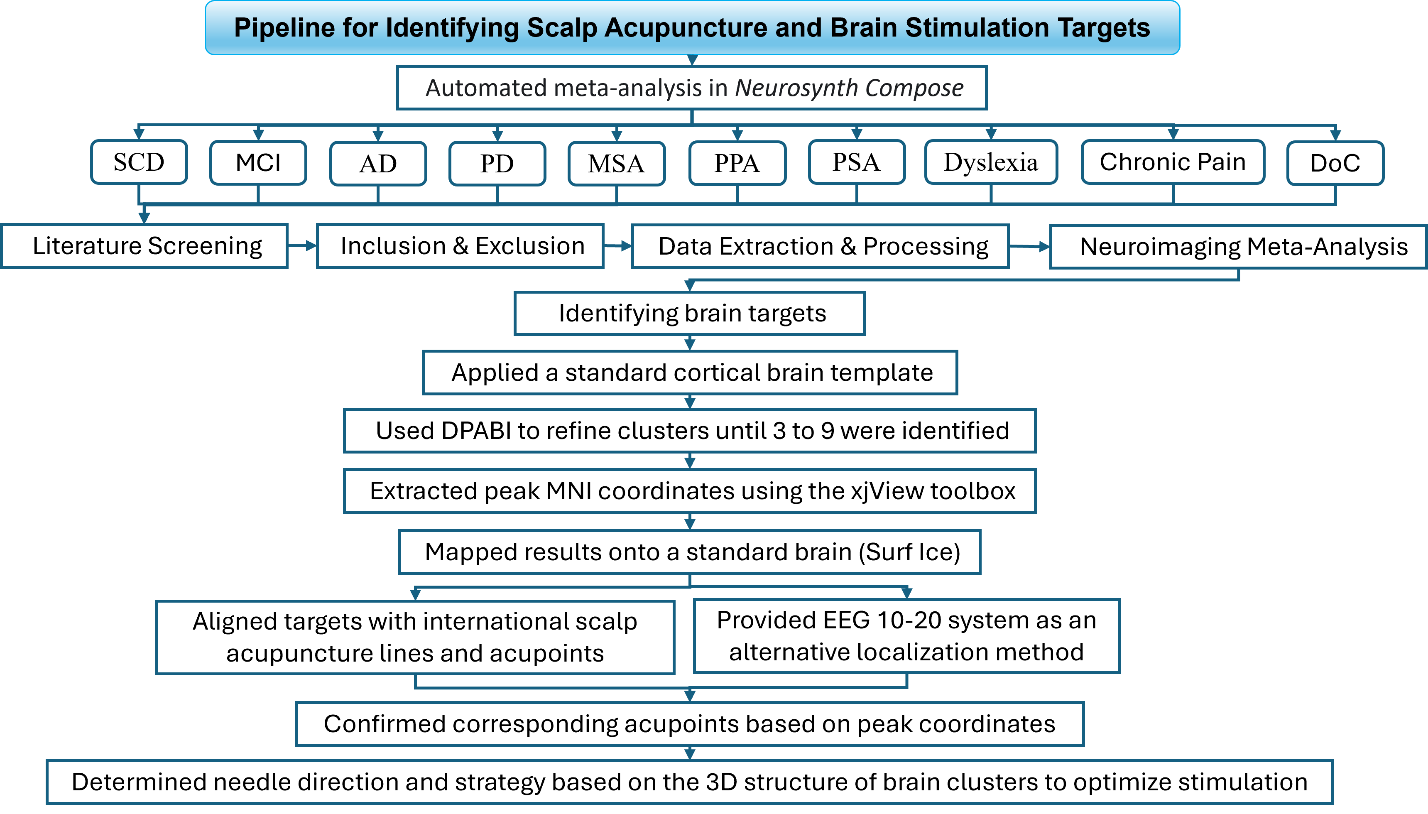
**

**Supplementary Figures 2 Reference for the localization of scalp acupuncture lines and acupoints**

**
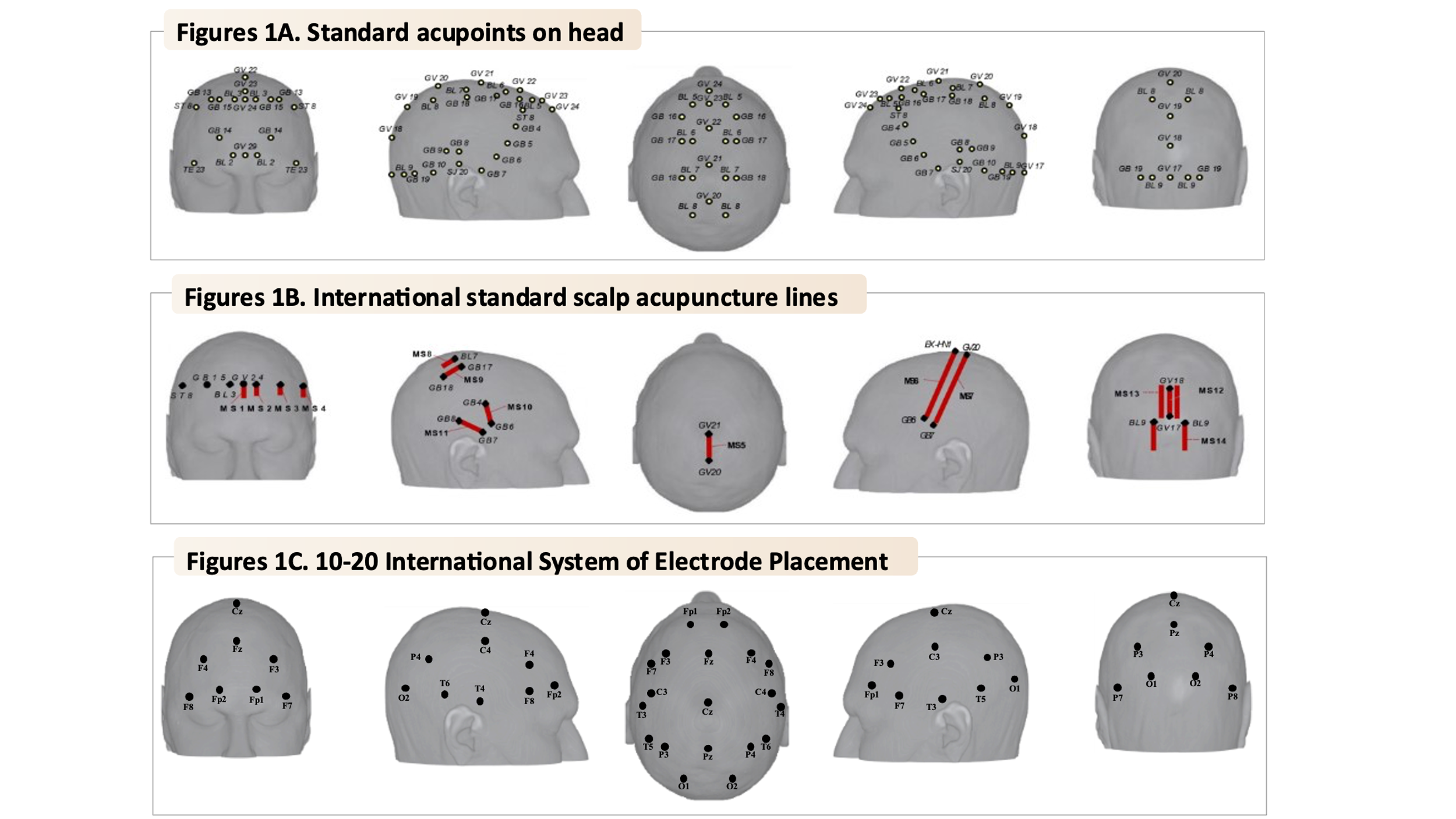
**

**Supplementary Table 1 Targets of Subjective Cognitive Decline (SCD)-related whole-brain regions identified from meta-analysis**

| **Targets** | **Number of Voxels** | **T value** | **Peak MNI** | | | **Corresponding brain area** | **Brain areas of the cluster** |
| --- | --- | --- | --- | --- | --- | --- | --- |
|  |  |  | **X** | **Y** | **Z** |  |  |
| SCD-01 | 839 | 5.97 | -22 | -28 | -22 | Parahippocampa Gyrus | Limbic Lobe |
|  |  |  |  |  |  |  | Parahippocampa Gyrus |
|  |  |  |  |  |  |  | Hippocampus_L |
|  |  |  |  |  |  |  | Thalamus_L |
|  |  |  |  |  |  |  | Amygdala_L |
| SCD-02 | 107 | 3.41 | 26 | -4 | -34 | Hippocampus_R | Limbic Lobe |
|  |  |  |  |  |  |  | Parahippocampa Gyrus |
|  |  |  |  |  |  |  | Amygdala_R |
|  |  |  |  |  |  |  | Hippocampus_R |
| SCD-03 | 32 | 2.57 | -58 | -18 | -20 | Temporal_Mid_L | Temporal_Mid_L |
| SCD-04 | 43 | 3.41 | -52 | 6 | -12 | Temporal_Pole_Sup_L | Temporal_Pole_Sup_L |
| SCD-05 | 44 | 4.25 | -52 | -58 | -2 | Temporal_Mid_L | Middle Temporal Gyrus |
|  |  |  |  |  |  |  | Temporal_Mid_L |
|  |  |  |  |  |  |  | Temporal_Inf_L |
| SCD-06 | 38 | 3.41 | 32 | 54 | -2 | Frontal_Mid_2_R | Frontal_Sup_2_R |
| SCD-07 | 30 | 3.41 | 36 | 20 | 0 | Insula_R | Insula_R |
| SCD-08 | 247 | 4.25 | 46 | 34 | 19 | Frontal_Inf_Tri_R | Frontal_Inf_Tri_R |
|  |  |  |  |  |  |  | Frontal_Mid_2_R |
|  |  |  |  |  |  |  | Inferior Frontal Gyrus |
| SCD-09 | 39 | 2.57 | -46 | 2 | 24 | Precentral_L | Precentral_L |
|  |  |  |  |  |  |  | Inferior Frontal Gyrus |
| SCD-10 | 613 | 6.42 | -42 | -52 | 46 | Parietal_Inf_L | Parietal_Inf_L |
|  |  |  |  |  |  |  | Parietal_Sup_L |
|  |  |  |  |  |  |  | Angular_L |
|  |  |  |  |  |  |  | Precuneus_L |
| SCD-11 | 185 | 3.41 | 48 | -38 | 36 | SupraMarginal_R | Parietal_Inf_R |
|  |  |  |  |  |  |  | SupraMarginal_R |
| SCD-12 | 110 | 4.25 | 24 | -62 | 62 | Parietal_Sup_R | Parietal_Sup_R |

Brain Region Reporting Based on the AAL3 (Automated Anatomical Labeling 3) Template.

**Supplementary Table 2 Targets of mild cognitive impairment (MCI)-related whole-brain regions identified from meta-analysis**

| **Targets** | **Number of Voxels** |  | **Peak MNI** | | | **Corresponding brain area** | **Brain areas of the cluster** |
| --- | --- | --- | --- | --- | --- | --- | --- |
|  |  |  | **X** | **Y** | **Z** |  |  |
| MCI-01 | 2247 | 12.75 | -26 | -26 | -18 | Hippocampus_L | Hippocampus_L |
|  |  |  |  |  |  |  | ParaHippocampal_L |
|  |  |  |  |  |  |  | Temporal Lobe |
|  |  |  |  |  |  |  | Amygdala_L |
|  |  |  |  |  |  |  | Thalamus_L |
| MCI-02 | 2376 | 13.18 | 22 | -10 | -18 | Hippocampus_R | Hippocampus_R |
|  |  |  |  |  |  |  | ParaHippocampal_R |
|  |  |  |  |  |  |  | Amygdala_R |
|  |  |  |  |  |  |  | Insula_R |
| MCI-03 | 248 | 5.64 | 54 | -4 | -22 | Temporal_Mid_R | Temporal_Mid_R |
|  |  |  |  |  |  |  | Temporal_Sup_R |
| MCI-04 | 839 | 5.90 | -56 | -6 | -16 | Temporal_Mid_L | Temporal_Mid_L |
|  |  |  |  |  |  |  | Occipital Lobe_L |
|  |  |  |  |  |  |  | Temporal_Inf_L |
| MCI-05 | 378 | 5.90 | -32 | 20 | -4 | Insula_L | Insula_L |
|  |  |  |  |  |  |  | Inferior Frontal Gyrus_L |
| MCI-06 | 822 | 6.17 | 0 | 44 | -4 | ACC_pre_L | ACC_pre_L |
|  |  |  |  |  |  |  | Medial Frontal Gyrus_bi |
|  |  |  |  |  |  |  | Frontal_Med_Orb_bi |
| MCI-07 | 375 | 6.44 | 40 | 20 | 0 | Insula_R | Insula_R |
|  |  |  |  |  |  |  | Inferior Frontal Gyrus_R |
| MCI-08 | 5053 | 11.35 | 2 | -50 | 26 | Cingulate_Post_R | Precuneus_bi |
|  |  |  |  |  |  |  | Posterior Cingulate_bi |
| MCI-09 | 1450 | 6.17 | 40 | -68 | 28 | Occipital_Mid_R | Angular_R |
|  |  |  |  |  |  |  | Inferior Parietal Lobule_R |
|  |  |  |  |  |  |  | Parietal_Inf_R |
| MCI-10 | 358 | 5.64 | 36 | 38 | 22 | Frontal_Mid_2_R | Frontal_Mid_2_R |
|  |  |  |  |  |  |  | Frontal_Inf_Tri_R |
| MCI-11 | 821 | 9.05 | -46 | 12 | 30 | Precentral_L | Frontal_Inf_Oper_L |
|  |  |  |  |  |  |  | Frontal_Inf_Tri_L |
|  |  |  |  |  |  |  | Precentral_L |
| MCI-12 | 243 | 5.11 | 6 | 28 | 32 | Cingulate_Mid_R | Cingulate_Mid_bi |
| MCI-13 | 248 | 5.37 | -24 | 18 | 52 | Frontal_Sup_2_L | Frontal_Mid_2_L |
|  |  |  |  |  |  |  | Frontal_Sup_2_L |
| MCI-14 | 57 | 4.31 | 28 | 18 | 50 | Frontal_Sup_2_R | Frontal_Sup_2_R |
|  |  |  |  |  |  |  | Frontal_Mid_2_R |
| MCI-15 | 34 | 3.78 | -2 | 6 | 50 | Supp_Motor_Area_L | Supp_Motor_Area_L |

Brain Region Reporting Based on the AAL3 (Automated Anatomical Labeling 3) Template.

**Supplementary Table 3 Targets of Alzheimer’s disease (AD) -related whole-brain regions identified from meta-analysis**

| **Targets** | **Number of Voxels** | **T value** | **Peak MNI** | | | **Corresponding brain area** | **Brain areas of the cluster** |
| --- | --- | --- | --- | --- | --- | --- | --- |
|  |  |  | **X** | **Y** | **Z** |  |  |
| AD-01 | 1982 | 11.54 | -26 | -16 | -20 | Hippocampus_L | Hippocampus_L  ParaHippocampal_L  Fusiform_L  Amygdala_L |
| AD-02 | 1881 | 11.26 | 24 | -10 | -18 | Hippocampus_R | Hippocampus_R  ParaHippocampal_R  Amygdala_R  Temporal Lobe |
| AD-03 | 251 | 5.14 | -58 | -30 | -12 | Temporal_Mid_L | Temporal_Mid_L  Temporal_Inf_L |
| AD-04 | 51 | 4.54 | 58 | -14 | -14 | Temporal_Mid_R | Temporal_Mid_R |
| AD-05 | 422 | 5.44 | -40 | -72 | -8 | Occipital_Inf_L | Occipital_Inf_L Temporal_Inf_L  Temporal_Mid_L |
| AD-06 | 1009 | 6.92 | -32 | 24 | 0 | Insula_L | Insula_L  Frontal_Inf_Tri_L  Frontal_Inf_Oper_L |
| AD-07 | 177 | 5.14 | 46 | 6 | 2 | Insula_R | Insula_R |
| AD-08 | 165 | 4.54 | 2 | 50 | 8 | ACC_pre_L | Anterior Cingulate Medial Frontal Gyrus |
| AD-09 | 3203 | 13.59 | -2 | -50 | 26 | Cingulate_Post_L | Posterior Cingulate Precuneus |
| AD-10 | 1220 | 8.39 | -48 | -66 | 28 | Angular_L | Angular_L  Middle Temporal Gyrus |
| AD-11 | 809 | 6.92 | 48 | -60 | 30 | Angular_R | Angular_R |
|  |  |  |  |  |  |  | Middle Temporal Gyrus |

Brain Region Reporting Based on the AAL3 (Automated Anatomical Labeling 3) Template.

**Supplementary Table 4 Targets of Parkinson’s Disease (PD) related whole-brain regions identified from meta-analysis**

| **Targets** | **Number of Voxels** | **T value** | **Peak MNI** | | | **Corresponding brain area** | **Brain areas of the cluster** |
| --- | --- | --- | --- | --- | --- | --- | --- |
|  |  |  | **X** | **Y** | **Z** |  |  |
| PD-01 | 195 | 4.75 | 28 | -60 | -24 | Cerebellum_6_R | Right Cerebellum |
| PD-02 | 8635 | 12.35 | -28 | -4 | 2 | Putamen_L | Putamen_bi  Thalamus_bi  Insula_bi  Caudate_bi  Pallidum_bi  Parahippocampa_bi  Amygdala_bi |
| PD-03 | 33 | 3.64 | -62 | -26 | 10 | Temporal_Sup_L | Temporal_Sup_L |
| PD-04 | 2800 | 6.99 | 48 | 6 | 28 | Precentral_R | Precentral_R  Frontal_Mid_2_R  Postcentral_R  Inferior Frontal Gyrus  Frontal_Sup_2_R  SupraMarginal_R |
| PD-05 | 47 | 4.3 | -36 | 46 | 12 | Frontal_Mid_2_L | Frontal_Mid_2_L |
| PD-06 | 78 | 3.85 | -4 | -48 | 24 | Cingulate_Post_L | Posterior Cingulate |
| PD-07 | 3840 | 9.16 | -34 | -24 | 56 | Precentral_L | Precentral_L  Postcentral_L  Parietal_Inf_L  Parietal_Sup_L  Angular_L  Frontal_Sup_2_L  Frontal_Mid_2_L  Frontal_Inf_Oper_L |
| PD-08 | 2902 | 12.04 | 2 | 8 | 54 | Supp_Motor_Area_R | Supp_Motor_Area_bi  Cingulate_Mid_bi |
| PD-09 | 150 | 4.52 | 8 | -70 | 50 | Precuneus_R | Precuneus_bi |

Brain Region Reporting Based on the AAL3 (Automated Anatomical Labeling 3) Template.

**Supplementary Table 5 Targets of Multiple System Atrophy (MSA)-related whole-brain regions identified from meta-analysis**

| **Targets** | **Number of Voxels** | **T value** | **Peak MNI** | | | **Corresponding brain area** | **Brain areas of the cluster** |
| --- | --- | --- | --- | --- | --- | --- | --- |
|  |  |  | **X** | **Y** | **Z** |  |  |
| MSA-01 | 10604 | 4.72 | 42 | -66 | -42 | Cerebellum_Crus2_R | Cerebellum  Brainstem  Medulla  Lentiform Nucleus  Extra-Nuclear  Putamen  Pyramis  Thalamus  Pons  Pallidum  Fusiform  Vermis  Insula |
| MSA-02 | 33 | 1.29 | -44 | -66 | -46 | Cerebellum_Crus2_L | Cerebellum_Crus2_L  Pyramis  Cerebellar Tonsil |
| MSA-03 | 42 | 1.29 | 4 | 38 | -32 | brodmann area 11 | Right Cerebrum |
| MSA-04 | 2278 | 4.72 | 22 | 6 | -10 | Putamen_R | Putamen_R  Lentiform Nucleus  Extra-Nuclear  Insula_R  Pallidum_R  Parahippocampa Gyrus  Rolandic_Oper_R  Hippocampus_R  Precentral_R  Amygdala_R |
| MSA-05 | 68 | 1.29 | 58 | -36 | -14 | Temporal_Mid_R | Temporal_Mid_R |
| MSA-06 | 132 | 2.22 | -38 | 46 | -6 | Frontal_Mid_2_L | Frontal_Mid_2_L |
| MSA-07 | 36 | 1.29 | 36 | 42 | -4 | - | Frontal_Mid_2_R |
| MSA-08 | 227 | 2.22 | -50 | 22 | 2 | Frontal_Inf_Tri_L | Frontal_Inf_Tri_L  Frontal_Inf_Oper_L, |
| MSA-09 | 88 | 2.22 | -52 | -54 | 6 | Temporal_Mid_L | Temporal_Mid_L |
| MSA-10 | 30 | 1.29 | -24 | 38 | 12 | - | Frontal Lobe Sub-Gyral |
| MSA-11 | 57 | 2.22 | 40 | 24 | 22 | Frontal_Inf_Tri_R | Frontal_Inf_Tri_R |
| MSA-12 | 584 | 3.1 | 16 | 18 | 42 | - | Frontal Lobe Sub-Gyral |
| MSA-13 | 43 | 1.29 | -14 | -68 | 30 | Precuneus_L | Precuneus_L |
| MSA-14 | 108 | 2.22 | -2 | 38 | 32 | Frontal_Sup_Medial_L | Frontal_Sup_Medial_L |
| MSA-15 | 57 | 2.22 | -12 | -32 | 34 | Cingulate_Mid_L | Cingulate_Mid_L |
| MSA-16 | 416 | 3.1 | -34 | 28 | 38 | Frontal_Mid_2_L | Frontal_Mid_2_L  Frontal_Sup_2_L |
| MSA-17 | 146 | 2.22 | -4 | -10 | 40 | Cingulate_Mid_L | Cingulate_Mid_L |
| MSA-18 | 72 | 1.29 | 44 | -62 | 38 | Angular_R | Angular_R |
| MSA-19 | 440 | 3.1 | -30 | -56 | 52 | Parietal_Inf_L | Parietal_Inf_L  Parietal_Sup_L |

Brain Region Reporting Based on the AAL3 (Automated Anatomical Labeling 3) Template.

**Supplementary Table 6 Targets of Post-Stroke Aphasia (PSA)-related whole-brain regions identified from meta-analysis**

| **Targets** | **Number of Voxels** | **T value** | **Peak MNI** | | | **Corresponding brain area** | **Brain areas of the cluster** |
| --- | --- | --- | --- | --- | --- | --- | --- |
|  |  |  | **X** | **Y** | **Z** |  |  |
| PSA-01 | 12593 | 9.34 | -46 | 22 | 20 | Frontal_Inf_Tri_L | Temporal_Mid_L  Frontal_Inf_Tri_L  Temporal_Sup_L  Sub-lobar_L  Insula_L  Frontal_Inf_Oper_L  Precentral_L  Angular_L  Frontal_Inf_Orb_2_L  Temporal_Inf_L  Postcentral_L  Occipital_Mid_L  Heschl_L  Frontal_Mid_2_L  Temporal_Pole_Sup_L  OFCpost_L  SupraMarginal_L  Parietal_Inf_L |
| PSA-02 | 462 | 4.98 | 32 | 18 | -4 | Insula_R | Insula_R |
| PSA-03 | 234 | 3.93 | 52 | -34 | 6 | Temporal_Sup_R | Temporal_Sup_R |
| PSA-04 | 159 | 3.93 | 50 | 28 | 16 | Frontal_Inf_Tri_R | Frontal_Inf_Tri_R  Frontal_Mid_2_R |
| PSA-05 | 233 | 3.39 | -18 | 56 | 12 | Frontal_Sup_2_L | Frontal_Sup_2_L Frontal_Mid_2_L |
| PSA-06 | 42 | 2.28 | -16 | 0 | 6 | Pallidum_L | Lentiform Nucleus  Putamen_L  Pallidum_L |
| PSA-07 | 995 | 6.01 | -6 | 28 | 38 | Frontal_Sup_Medial_L | Frontal_Sup_Medial_L  Frontal_Sup_Medial_L  Cingulate_Mid_L |

Brain Region Reporting Based on the AAL3 (Automated Anatomical Labeling 3) Template.

**Supplementary Table 7 Targets Primary Progressive Aphasia (PPA)-related whole-brain regions identified from meta-analysis**

| **Targets** | **Number of Voxels** | **T value** | **Peak MNI** | | | **Corresponding brain area** | **Brain areas of the cluster** |
| --- | --- | --- | --- | --- | --- | --- | --- |
|  |  |  | **X** | **Y** | **Z** |  |  |
| PPA-01 | 9065 | 8.64 | -54 | -30 | 6 | Temporal_Sup_L | Temporal_Sup_L  Temporal_Mid_L  Temporal_Inf_L  Frontal_Inf _L  Fusiform_L  Precentral_L  Temporal_Pole_Sup_L  Angular_L  Parahippocampa Gyrus  Insula_L  Temporal_Pole_Mid_L  Hippocampus_L  Extra-Nuclear  Putamen  Postcentral_L  Amygdala_L |
| PPA-02 | 1262 | 5.58 | 38 | 22 | -6 | Insula_R | Insula_R  Frontal_Inf _R  Temporal_Mid_R  Temporal_Pole _R |
| PPA-03 | 50 | 2.19 | 14 | 10 | -2 | Putamen_R | Putamen_R |
| PPA-04 | 273 | 4.92 | -8 | 42 | 4 | ACC_pre_L | ACC_pre_L  Frontal_Sup_Medial_L |
| PPA-05 | 103 | 3.59 | -40 | 38 | 12 | Frontal_Inf_Tri_L | Frontal_Inf_Tri_L  Frontal_Mid_2_L |
| PPA-06 | 58 | 3.59 | -32 | -78 | 24 | Occipital_Mid_L | Occipital_Mid_L |
| PPA-07 | 68 | 2.90 | -10 | -56 | 28 | Precuneus_L | Precuneus_L  Cingulate_Post_L |
| PPA-08 | 272 | 4.27 | -4 | 10 | 40 | Cingulate_Mid_L | Cingulate_Mid_L  Supp_Motor_Area_L |
| PPA-09 | 112 | 3.59 | 44 | -8 | 44 | Precentral_R | Precentral_R |

Brain Region Reporting Based on the AAL3 (Automated Anatomical Labeling 3) Template.

**Supplementary Table 8 Targets of Dyslexia -related whole-brain regions identified from meta-analysis**

| **Targets** | **Number of Voxels** | **T value** | **Peak MNI** | | | **Corresponding brain area** | **Brain areas of the cluster** |
| --- | --- | --- | --- | --- | --- | --- | --- |
|  |  |  | **X** | **Y** | **Z** |  |  |
| Dys-01 | 30 | 5.32 | 28 | -60 | -26 | Cerebellum_6_R | Cerebellum_6_R |
| Dys-02 | 1018 | 12.97 | -42 | -52 | -16 | Fusiform_L | Fusiform_L  Temporal_Inf_L  Occipital_Inf_L  Middle Occipital Gyrus |
| Dys-03 | 45 | 6.18 | 42 | -64 | -14 | Occipital_Inf_R | Occipital_Inf_R |
| Dys-04 | 3211 | 15.75 | -46 | 6 | 28 | Frontal_Inf_Oper_L | Frontal_Inf_Oper_L  Frontal_Inf_Tri_L  Precentral_L  Insula_L  Sub-lobar  Frontal_Inf_Orb_2_L  Frontal_Mid_2_L  Temporal_Pole_Sup_L  Postcentral_L |
| Dys-05 | 203 | 6.61 | -26 | -94 | -5 | Occipital_Inf_L | Occipital_Inf_L  Occipital_Mid_L |
| Dys-06 | 408 | 8.30 | 34 | 22 | 0 | Insula_R | Insula_R  Frontal_Inf_Orb_2_R |
| Dys-07 | 2719 | 10.42 | -46 | -46 | 36 | Parietal_Inf_L | Parietal_Inf_L  Temporal_Mid_L  Temporal_Sup_L  SupraMarginal_L  Parietal_Sup_L  Angular_L |
| Dys-08 | 51 | 6.18 | 50 | 8 | 32 | Precentral_R | Precentral_R |
| Dys-09 | 935 | 11.27 | -2 | 10 | 52 | Supp_Motor_Area_L | Supp_Motor_Area_L Medial Frontal Gyrus  Supp_Motor_Area_R  Cingulate_Mid_bi |
| Dys-10 | 96 | 6.18 | 34 | -64 | 42 | Angular_R | Angular_R |

Brain Region Reporting Based on the AAL3 (Automated Anatomical Labeling 3) Template.

**Supplementary Table 9 Targets of Chronic Pain -related whole-brain regions identified from meta-analysis**

| **Targets** | **Number of Voxels** | **T value** | **Peak MNI** | | | **Corresponding brain area** | **Brain areas of the cluster** |
| --- | --- | --- | --- | --- | --- | --- | --- |
|  |  |  | **X** | **Y** | **Z** |  |  |
| CP-01 | 359 | 7.25 | -26 | -6 | -22 | Hippocampus_L | ParaHippocampal_L Hippocampus_L  Amygdala_L  Uncus |
| CP-02 | 2195 | 7.25 | -2 | 32 | 20 | ACC_sup_L | ACC_bi  Cingulate_Mid_bi  Frontal_Sup_Medial_bi  Frontal_Med_Orb__bi |
| CP-03 | 2265 | 9.30 | -40 | -18 | 10 | Insula_L | Insula_L  Rolandic_Oper_L  Postcentral_L  Temporal_Sup_L  Precentral Gyrus  Heschl_L  SupraMarginal_L  Frontal_Inf_Tri_L |
| CP-04 | 2440 | 10.72 | 38 | 10 | 2 | Insula_R | Insula_R  Rolandic_Oper_R  Putamen_R  Postcentral Gyrus  Precentral Gyrus  SupraMarginal_R  Frontal_Inf_Oper_R  Temporal_Sup_R  Putamen |
| CP-05 | 811 | 7.25 | -12 | -18 | 4 | Thal_IL_L | Thalamus(VL-bi,VPL_bi, MD_bi, PuA_bi) |
| CP-06 | 31 | 4.17 | -14 | 12 | -2 | Putamen_L | Putamen_L |
| CP-07 | 45 | 4.48 | 34 | 48 | 18 | Frontal_Sup_2_R | Frontal_Sup_2_R |
| CP-08 | 140 | 4.79 | -8 | -58 | 20 | Precuneus_L | Precuneus_bi  Cingulate_Post_bi |
| CP-09 | 207 | 5.72 | -4 | -18 | 38 | Cingulate_Mid_L | Cingulate_Mid_bi |
| CP-10 | 70 | 4.79 | -48 | -20 | 42 | Postcentral_L | Postcentral_L |
| CP-11 | 122 | 5.10 | -44 | -54 | 42 | Parietal_Inf_L | Parietal_Inf_L |
| CP-12 | 167 | 4.48 | 36 | -30 | 56 | Postcentral_R | Postcentral_R |

Brain Region Reporting Based on the AAL3 (Automated Anatomical Labeling 3) Template.

**Supplementary Table 10 Targets of Disorders of Consciousness (DoC)-related whole-brain regions identified from meta-analysis**

| **Targets** | | **Number of Voxels** | **T value** | **Peak MNI** | | | **Corresponding brain area** | **Brain areas of the cluster** |
| --- | --- | --- | --- | --- | --- | --- | --- | --- |
|  |  |  |  | **X** | **Y** | **Z** |  |  |
| DoC-01 | 1396 | 5.23 | 12 | 8 | -8 | N_Acc_R | Pallidum_R  Thal_VL_R  Putamen_R  ParaHippocampal_R  N_Acc_R  Caudate_R  Ventral Nucleus  Amygdala_R  Insula_R  Hippocampus_R | |
| DoC-02 | 219 | 4.16 | 50 | 10 | -20 | Temporal_Pole_Sup_R | Temporal_Pole_Sup_R  Temporal_Mid_R | |
| DoC-03 | 679 | 4.16 | -30 | 24 | -14 | OFCpost_L | Insula_L  Frontal_Inf_Orb_2_L  OFCpost_L  Rolandic_Oper_L  Frontal_Inf_Tri_L | |
| DoC-04 | 74 | 3.09 | -22 | -24 | -18 | Parahippocampa Gyrus | Parahippocampa Gyrus  Hippocampus_L | |
| DoC-05 | 294 | 4.16 | -12 | 4 | -10 | - | Sub-lobar  Lentiform Nucleus  Putamen_L  Pallidum_L  Amygdala_L | |
| DoC-06 | 346 | 4.16 | 6 | 44 | 0 | ACC_pre_R | ACC_pre_bi  Frontal_Med_Orb_bi | |
| DoC-07 | 57 | 2.10 | 36 | 54 | -4 | Frontal_Mid_2_R | Frontal_Mid_2_R  Frontal_Sup_2_R | |
| DoC-08 | 103 | 2.10 | 54 | -14 | 0 | Temporal_Sup_R | Temporal_Sup_R  Rolandic_Oper_R | |
| DoC-09 | 134 | 3.09 | -12 | -24 | 8 | Thal_PuM_L | Thalamus_L | |
| DoC-10 | 1192 | 7.04 | 0 | -58 | 28 | Precuneus_L | Precuneus_bi  Cingulate_Post_bi  Cingulate_Mid_L | |
| DoC-11 | 35 | 3.09 | -10 | 12 | 14 | Caudate_L | Caudate_L | |
| DoC-12 | 317 | 3.09 | -8 | 28 | 14 | Limbic Lobe | ACC_bi | |
| DoC-13 | 86 | 3.09 | -2 | -32 | 26 | Cingulate_Post_L | Cingulate_Mid_L  Cingulate_Post_L | |
| DoC-14 | 318 | 4.16 | -38 | -76 | 38 | Occipital_Mid_L | Angular_L,  Occipital_Mid_L  Parietal_Inf_L | |
| DoC-15 | 528 | 6.27 | 2 | 2 | 40 | Cingulate_Mid_R | Cingulate_Mid_bi | |
| DoC-16 | 42 | 2.10 | 48 | 12 | 30 | Frontal_Inf_Oper_R | Frontal_Inf_Oper_R  Frontal_Mid_2_R | |
| DoC-17 | 32 | 3.09 | 44 | -2 | 36 | Precentral_R | Precentral_R | |
| DoC-18 | 102 | 3.09 | 48 | -44 | 44 | SupraMarginal_R | Parietal_Inf_R  SupraMarginal_R | |
| DoC-19 | 219 | 4.16 | -24 | 14 | 52 | Frontal_Sup_2_L | Frontal_Mid_2_L  Frontal_Sup_2_L | |
| DoC-20 | 33 | 2.10 | 2 | -62 | 50 | Precuneus_R | Precuneus_R | |
| DoC-21 | 52 | 2.10 | -20 | 14 | 60 | Frontal_Sup_2_L | Frontal_Sup_2_L | |

Brain Region Reporting Based on the AAL3 (Automated Anatomical Labeling 3) Template.

**Supplementary Table 11 The international standard names for the acupoints used in these protocols**

| **WHO Standard Code** | **Pinyin (Romanized)** | **Japanese Name (Kanji, Reading)** | **Korean Name (Hangul, Reading)** |
| --- | --- | --- | --- |
| GB14 | Yangbai | 陽白 (ようはく, Yōhaku) | 양백 (양백, Yangbaek) |
| TE23 | Sizhukong | 絲竹空 (しちくくう, Shichikukū) | 사죽공 (사죽공, Sajukgong) |
| BL8 | Luque | 絡却 (らっきゃく, Rakkaku) | 락각 (락각, Ragag) |
| GB9 | Tianchong | 天衝 (てんしょう, Tenshō) | 천충 (천충, Cheonchung) |
| GB10 | Fubai | 浮白 (ふはく, Fuhaku) | 부백 (부백, Bubek) |
| GB8 | Shuaigu | 率谷 (そっこく, Sokkoku) | 솔곡 (솔곡, Solgok) |
| GB7 | Qubin | 曲鬢 (きょくびん, Kyokubin) | 곡빈 (곡빈, Gokbin) |
| GB4 | Hanyan | 頷厭 (がんえん, Gan'en) | 함염 (함염, Hamyeom) |
| GB5 | Xuanlu | 懸顱 (けんろ, Kenro) | 현로 (현로, Hyeonno) |
| GB16 | Muang | 目窓 (もくそう, Mokusō) | 목창 (목창, Mokchang) |
| GB17 | Zhengying | 正営 (せいえい, Sei'ei) | 정영 (정영, Jeongyeong) |
| SJ20 | Jiaosun | 角孫 (かくそん, Kakuson) | 각손 (각손, Gakson) |
| GB18 | Chengling | 承霊 (じょうれい, Jōrei) | 승령 (승령, Seungnyeong) |
| GB6 | Xuanli | 懸厘 (けんり, Kenri) | 현리 (현리, Hyeonri) |
| GV21 | Qianding | 前頂 (ぜんちょう, Zenchō) | 전정 (전정, Jeonjeong) |
| GV29 | Yinjiao | 齦交 (ぎんこう, Ginkō) | 은교 (은교, Eungyo) |
